# Ocean warming and acidification adjust inter- and intra-specific variability in the functional trait expression of polar invertebrates

**DOI:** 10.1038/s41598-024-65808-5

**Published:** 2024-07-01

**Authors:** Thomas J. Williams, Adam J. Reed, Lloyd S. Peck, Jasmin A. Godbold, Martin Solan

**Affiliations:** 1 School of Ocean and Earth Science, National Oceanography Centre Southampton, University of Southampton, Waterfront Campus, European Way, Southampton, SO14 3ZH UK; 2 British Antarctic Survey, NERC, High Cross, Madingley Road, Cambridge, CB3 0ET UK

**Keywords:** Bioturbation, Benthic, Plasticity, Climate futures, Polar front, Arctic, Antarctic, Marine biology, Behavioural ecology, Climate-change impacts, Phenology

## Abstract

Climate change is known to affect the distribution and composition of species, but concomitant alterations to functionally important aspects of behaviour and species-environment relations are poorly constrained. Here, we examine the ecosystem ramifications of changes in sediment-dwelling invertebrate bioturbation behaviour—a key process mediating nutrient cycling—associated with near-future environmental conditions (+ 1.5 °C, 550 ppm [pCO_2_]) for species from polar regions experiencing rapid rates of climate change. We find that responses to warming and acidification vary between species and lead to a reduction in intra-specific variability in behavioural trait expression that adjusts the magnitude and direction of nutrient concentrations. Our analyses also indicate that species behaviour is not predetermined, but can be dependent on local variations in environmental history that set population capacities for phenotypic plasticity. We provide evidence that certain, but subtle, aspects of inter- and intra-specific variation in behavioural trait expression, rather than the presence or proportional representation of species per se, is an important and under-appreciated determinant of benthic biogeochemical responses to climate change. Such changes in species behaviour may act as an early warning for impending ecological transitions associated with progressive climate forcing.

## Introduction

Narratives of the ecological consequences of climate change often centre on biodiversity, food-web structure and productivity^1–3^, rather than the ecological consequences of alternative outcomes that typically form the prelude to compositional restructuring and/or altered levels of biodiversity^4,5^. Species responses to a changing climate can include avoidance through dispersal^6^, acclimation through phenotypic plasticity^7,8^, including adjustments to physiological regulation^9^, and adaptation through genetic modification^10^. However, these alternative strategies are not always viable or, when available, are not necessarily equally weighted as an effective means of response^11^. Indeed, in areas of greater risk from environmental change, such as those at higher latitudes, opportunities for dispersal (including instances of > 40 days^12^) and adaptation are often limited due to local evolutionary history and ecology^13^, meaning that phenotypic plasticity becomes the de facto mechanism of response^14^. For organisms with very long generation times, as is common in polar regions^15,16^, behavioural acclimatisation can maximise an individual’s chance of survival^17,18^ in advance of genetic adaptation^19^, unless fecundity is sufficient to increase the likelihood that gene adaptations arise in the population^20^. Previous work mainly focuses on invertebrate physiological plasticity in relation to ocean warming^21,22^ and acidification^23–25^, with less emphasis on behavioural plasticity^26^, even though changes in behaviour often form the first practical response to altered environmental context^27,28^ and can have consequences for other ecosystem attributes^29^. Consequently, the specifics of how and when climate related change affects the way in which species behaviour modifies ecosystem functioning is under-appreciated^30,31^.

The activities of sediment-dwelling invertebrates redistribute pore water fluids and sediment particles, ultimately affecting carbon and nutrient cycles^32,33^. It follows, therefore, that any directional change in species behaviour or trait expression will have important consequences for ecosystem process and function^34^. Such changes, although species and context dependent^35–37^, reflect individual responses to changing circumstances that may maintain^38^, reduce^35^ or enhance^39–41^ functioning, making it difficult to generalise species contributions to alterations in ecosystem properties. Disentangling these effects is frustrated by the fact that changes in behaviour are also accompanied by compensatory responses^42,43^ that affect dominance patterns^44,45^, and other factors, which can partially, or wholly, offset functional responses to forcing^46^. Nevertheless, field observations show that a shift in the type and amount of faunal activity can lead to environmental transitions^3^ that exert a disproportionate influence on ecosystem properties and functioning over and above the effects caused by changes in species diversity^47,48^ and composition^45,49^. It is important to note, however, that although flexible behavioural strategies can improve short-term fitness^50,51^, any associated functional consequences^52,53^ may not materialise until much later and can be hard to distinguish from other temporal changes in the system^54^.

We anticipated that changes in species behaviour will be more pronounced in regions of fast paced climate change^3,55^, as genetic and other coping mechanisms are less likely to be enacted in time. We speculated, given the closure of dispersal and adaptation as viable options, that adjustments to individual behaviour would dominate species responses to change^56^ at higher latitudes. Here, using sediment-dwelling invertebrate species obtained from the Arctic Barents Sea (the bivalve *Astarte crenata,* sea star *Ctenodiscus crispatus* and polychaete *Cistenides hyperborea*) and Antarctic Peninsula (the protobranch *Aequiyoldia eightsi* and bivalve *Laternula elliptica*), two areas currently experiencing amplified climate change^57,58^, we explore the combined effects of near-term ocean warming (+ 1.5 °C) and elevated levels of atmospheric carbon dioxide (550 ppm [CO_2_]) on aspects of species behaviour known to influence biogeochemical cycling. As we anticipate that the direction and magnitude of change in behaviour will diverge between species^4,59,60^, we also include individuals of *Astarte crenata* and *Ctenodiscus crispatus* from two locations within the Barents Sea that contrast in temperature and sea ice dynamics; here, our expectation is that individual species responses will be in line with previous observations^3^, but will be more pronounced when species are from locations experiencing narrower environmental variation. We use these data to demonstrate the importance of behavioural change and compensatory mechanisms, including numeric and/or biomass increases and performance enhancement^42,43^, in moderating how benthic environments respond to external forcing. We show, for five species of polar benthic invertebrates, that the ability to modify behaviour in the face of climatic forcing does not guarantee that species contributions will remain unchanged. Our findings emphasise the importance of context-dependency and have implications for the functional contributions of populations facing climate change, their capacity to adapt in the face of further environmental transitions, and suggest that the onset of phenotypic expression may serve as an early warning for impending ecological change.

## Results

We find evidence that individual movement and burial behaviour, sediment particle reworking activity, burrow ventilation activity, and associated nutrient concentrations at the sediment–water interface, can be dependent on environmental condition (ambient climate treatment vs future climate treatment of + 1.5 °C and 550 ppm [CO_2_]), location, and species identity (Supplementary Models S1 to S29). However, observed effects seldom form full factorial interactions between the three dependent variables (8 of 29 models). Despite observing mortalities in the bivalve *Astarte crenata* (2 individuals, 1 from each climate), the sea star *Ctenodiscus crispatus* (4 individuals, 3 ambient and 1 future climate), and the polychaete *Cistenides hyperborea* (1 ambient climate), it was possible to relate our response variables in ecosystem process (sediment particle reworking: surface boundary roughness, median mixed depth and maximum mixed depth; burrow ventilation activity) and functioning (nutrient concentrations: ammonium, nitrite, nitrate and phosphate) to species behaviour (individual movement: response time; burial behaviour: burial time) in all aquaria. We find no evidence that differences in mortality (assessed using total biomass as a random effect) affects our response variables.

### Effects on individual behaviour

**Figure 1:**
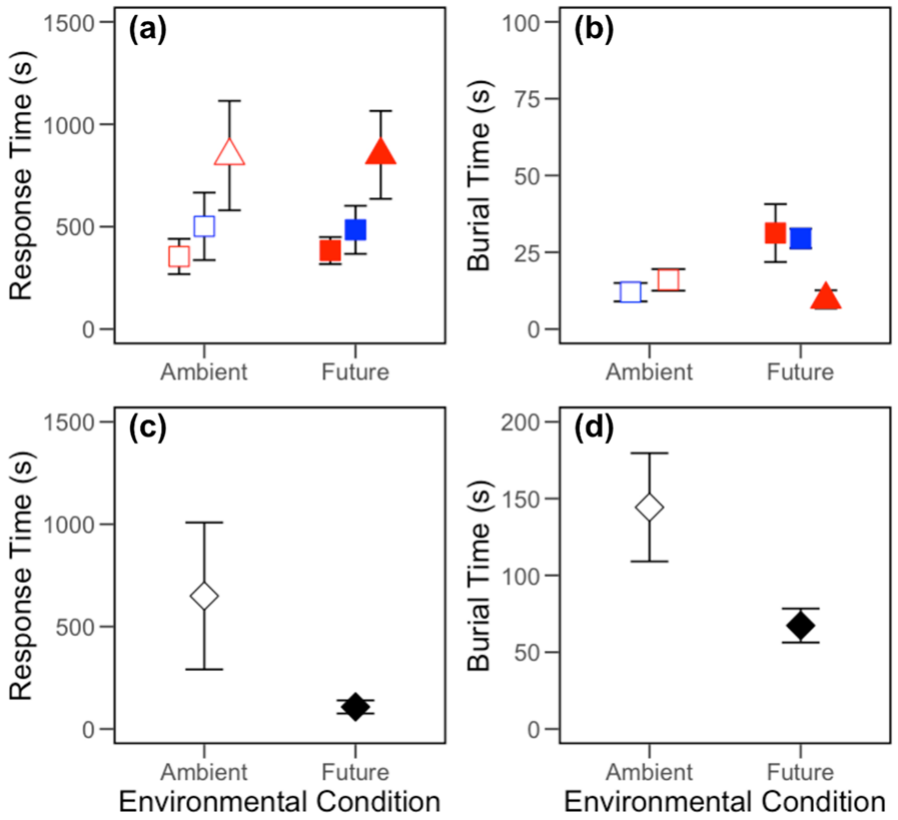
The effects of species identity, location and environmental condition (ambient, open symbols; future, closed symbols) on (**a**,**c**) mean (± s.e.) response time and (**b**,**d**) mean (± s.e.) burial time for *Ctenodiscus crispatus* (□) and *Cistenides hyperborea* (△) obtained from station B13 (red) and B16 (blue) in the Barents Sea and *Aequiyoldia eightsi* (◇) obtained from Rothera Point (black). Individuals of *C. hyperborea* did not rebury under ambient conditions.

All individuals of *C. crispatus* (n_T_ = 18) initiated movement within 60 min, with 16 individuals completing reburial, but we found no evidence that response time was affected by environmental condition, location or their interaction (intercept only model: L-ratio = 1.420, d.f. = 1, *p* = 0.234; Fig. 1a). However, response times were less variable between individuals from station B13 (coefficient of variation, CV = 34.5%) relative to individuals from station B16 (CV = 62.9%). Regardless of location, mean burial time of *C. crispatus* was influenced by environmental condition (F_[1,12]_ = 5.285, *p* < 0.05), with reburial time halving under future conditions (Fig. 1b). For *C. hyperborea*, 9 individuals (n_T_ = 11) responded within 60 min, with comparable response rates across both environmental conditions (F_[1,7]_ < 0.001, *p* = 0.992; Fig. 1a). However, no individuals reburied under ambient conditions and an insufficient number of individuals (n = 3) reburied within 60 min under future conditions for reliable statistical analysis. For *A. eightsi*, response time was not dependent on environmental condition (intercept only model, L-ratio = 2.277, d.f. = 1, *p* = 0.131; Fig. 1c), despite a substantial reduction in intra-specific variability under future conditions (CV: ambient, 95.7%; future, 51.5%). Burial time for *A. eightsi* was weakly dependent on environmental condition (L-ratio = 3.5943, d.f. = 1, *p* = 0.0580), despite a reduction in intra-specific variability (CV: ambient = 42.3%, future = 28.4%) and burial time (Fig. 1d). We found no effect of biomass as a random factor in any of these models.

### Effects on ecosystem process

**Figure 2:**
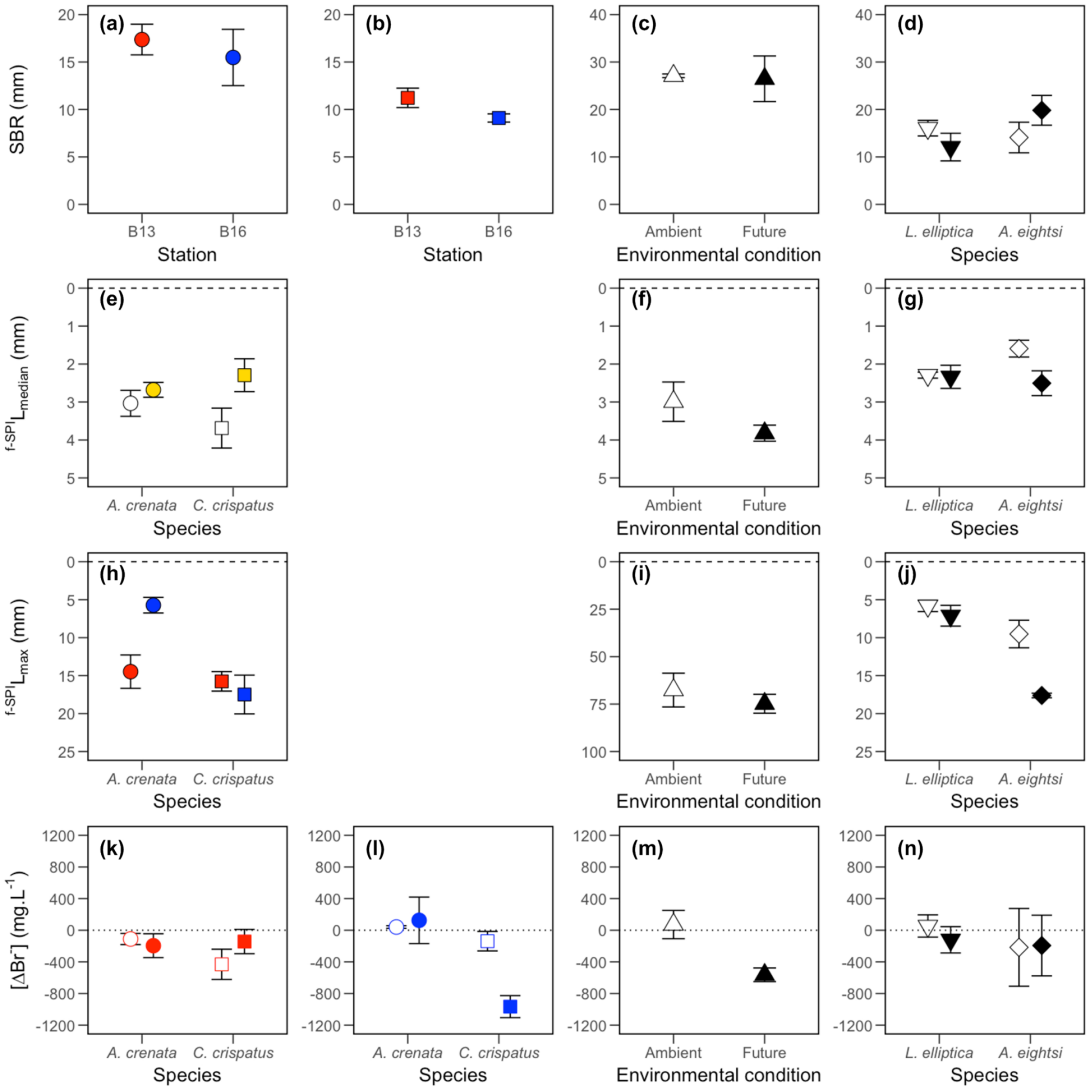
The effects of species identity, location and environmental condition (ambient, open symbols; future, closed symbols) on (mean ± s.e.) (**a**–**d**) SBR (mm), (**e**–**g**) ^f-SPI^L_median_ (mm), (**h**–**j**) ^f-SPI^L_max_ (mm) and (**k**,**l**–**n**) [∆Br^−^] (mg L^−1^) in mesocosms containing (**a**,**b**,**d**,**h**,**k**,**l**) *Astarte crenata* (○) or *Ctenodiscus crispatus* (□) from station B13 (red), B16 (blue) or both locations combined (gold), (**c**,**f**,**i**,**m**) mesocosms containing *Cistenides hyperborea* (△) obtained from station B13 and (**d**,**g**,**j**,**n**) mesocosms containing *Aequiyoldia eightsi* (◇) or *Laternula elliptica* (▽) obtained from Rothera Point. For ∆[Br^﻿−^], negative values indicate increased bioirrigation. Sediment profile images and associated luminophore distribution profiles are presented in Supplementary Figs. S8–S11.

Surface boundary roughness (SBR) in the presence of *A. crenata* and *C. crispatus* (Fig. 2a–b) was dependent on the independent effects of species (L-ratio = 10.056, d.f. = 1, *p* < 0.01) and location (L-ratio = 4.010, d.f. = 1, *p* < 0.05), but not environmental condition (L-ratio = 3.238, d.f. = 1, *p* = 0.072). For *C. hyperborea*, we also found no evidence that SBR was affected by changes in environmental condition (L-ratio = 0.025, d.f. = 1, *p* = 0.8740) despite an increase in intra-specific variability under future conditions (CV: ambient, 2.5%; future, 31.4%; Fig. 2c). For *A. eightsi* and *L. elliptica*, we found no effect of environmental condition, species identity, or their interactions, on SBR (F_[1,8]_ = 3.005, *p* = 0.121; Fig. 2d).

The median mixed depth of particle reworking (^f-SPI^L_med_) for *A. crenata* and *C. crispatus* was dependent on the independent effect of environmental condition (F_[1,18]_ = 5.2018, *p* < 0.05; Fig. 2e). However, there was no effect of environmental condition on ^f-SPI^L_med_ for *C. hyperborea* (L-ratio = 0.338, d.f. = 1, *p* = 0.126; Fig. 2f) or for *A. eightsi* and *L. elliptica* (F_[1,8]_ = 2.955, *p* = 0.124; Fig. 2g). In contrast, maximum mixed depth (^f-SPI^L_max_) was dependent on a species identity × location interaction for *A. crenata* and *C. crispatus* (F_[1,20]_ = 7.8123, *p* < 0.05), with species identity (*ω*^2^ = 0.537) more influential than location (*ω*^2^ = 0.316). Specifically, ^f-SPI^L_max_ was deeper in aquaria containing *C. crispatus* from station B16 than it was in aquaria containing *A. crenata* from station B16 and, to a lesser extent, station B13 (Fig. 2h). For *C. hyperborea,* ^f-SPI^L_max_ was not dependent on environmental condition (intercept only model: ^f-SPI^L_max_, L-ratio = 0.695, d.f. = 1, *p* = 0.405), but there was some evidence for a reduction in intra-specific variability between treatments (CV: ambient, 22.8%; future, 11.5%; Fig. 2i). In contrast, we found that ^f-SPI^L_max_ for *A. eightsi* and *L. elliptica* was dependent on an environmental condition × species identity interaction (F_[1,8]_ = 7.962, *p* < 0.05), with species identity (*ω*^2^ = 1.103) more influential than environmental condition (*ω*^2^ = 0.907). Specifically, ^f-SPI^L_max_ was deeper for *A. eightsi* relative to *L. elliptica*, with a larger difference observed under future conditions (Fig. 2j).

The burrow ventilation activity ([∆Br^−^]) of *A. crenata* and *C. crispatus* was dependent on an environmental condition × location × species identity interaction (F_[1,16]_ = 7.910, *p* < 0.05), with species identity the most influential independent variable (*ω*^2^ = 0.678), followed by location (*ω*^2^ = 0.481) and environmental condition (*ω*^2^ = 0.376). In individuals from station B13, irrespective of species identity, [∆Br^−^] was unchanged by environmental condition (Fig. 2k). However, whilst [∆Br^−^] for *A. crenata* from station B16 was negligible ([∆Br^−^] values were positive) in both ambient and future environmental conditions, [∆Br^−^] for *C. crispatus* increased sevenfold (values more negative) under the future environmental condition (Fig. 2l). [∆Br^−^] of *C. hyperborea* was also affected by environmental condition (L-ratio = 5.879, d.f. = 1, *p* < 0.05) with an increase in burrow ventilation activity under future environmental conditions (Fig. 2m). In contrast, there was no effect of environmental condition or species identity on [∆Br^−^] for *A. eightsi* and *L. elliptica* (intercept only; L-ratio = 0.764, d.f. = 1, *p* = 0.382; Fig. 2n), but we did observe a reduction in intra-specific variability between treatments (CV: ambient, 713%; future, 293%).

### Effects on ecosystem functioning

**Figure 3:**
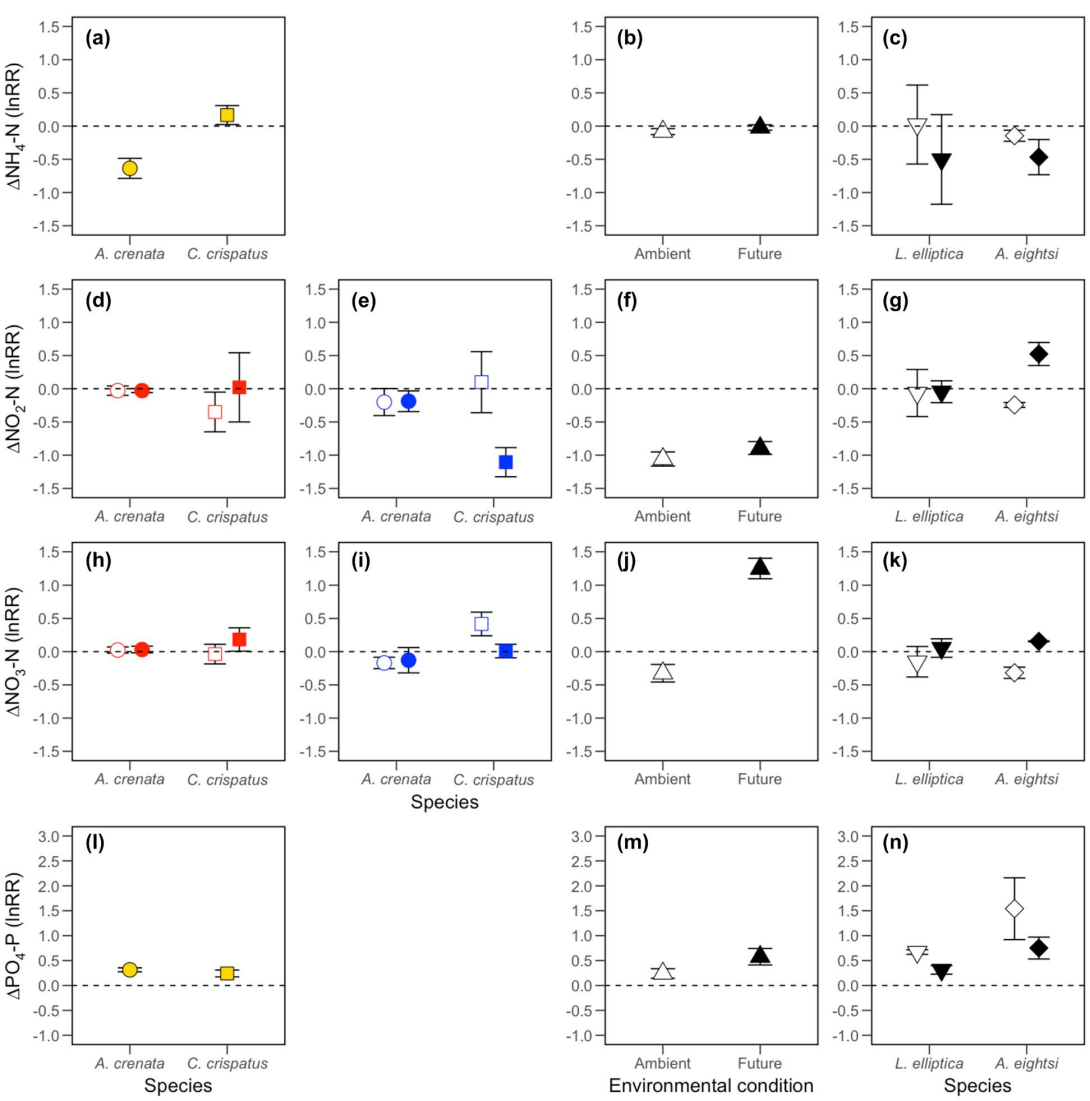
The effects of species identity, location and environmental condition (ambient, open symbols; future, closed symbols) on (mean ± s.e.) effect size of nutrient concentrations (lnRR) over the experimental period as indicated by (**a**–**c**) [NH_4_-N], (**d**–**g**) [NO_2_-N], (**h**–**k**) [NO_3_-N] and (**l**–**n**) [PO_4_-P] in mesocosms containing (**a**,**d**,**e**,**h**,**i**,**l**) *Astarte crenata* (○) or *Ctenodiscus crispatus* (□) from station B13 (red), B16 (blue) or both (gold), (**b**,**f**,**j**,**m**) mesocosms containing *Cistenides hyperborea* (△) obtained from station B13 and (**c**,**g**,**k**,**n**) mesocosms containing *Aequiyoldia eightsi* (◇) or *Laternula elliptica* (▽) obtained from Rothera Point. A positive effect size indicates an increase in nutrient release from the sediment into the water column over the experimental period, while a negative effect size signifies an increase in the uptake of nutrients from the water column into the sediment.

Our analyses reveal that, for *A. crenata* and *C. crispatus*, ammonium ([NH_4_-N]) was influenced by the independent effect of species identity (F_[1,22]_ = 14.951, *p* < 0.0001), with positive log response ratios (lnRRs) in aquaria containing *C. crispatus* and negative lnRRs in aquaria containing *A. crenata* (Fig. 3a). We find that the effect size for [NH_4_-N] is not dependent on environmental condition in the presence of *C. hyperborea* (intercept only models: [NH_4_-N], F_[1.4]_ = 1.047, *p* = 0.364; Fig. 3b), *A. eightsi* or *L. elliptica* (intercept only model, L-ratio = 0.009, d.f. = 1, *p* = 0.925; Fig. 3c). For nitrite ([NO_2_-N]), whilst there is evidence of a weak dependence on environmental condition in the presence of *L. elliptica* and *A. eightsi* (L-ratio = 3.532, d.f. = 1, *p* = 0.060; Fig. 3g), the effect size of [NO_2_-N] in the presence of *A. crenata* and *C. crispatus* was dependent on an environmental condition × location × species identity interaction (L-ratio = 4.629, d.f. = 1, *p* < 0.05). For the latter, model coefficients revealed that location was most influential (L-ratio = 7.714, d.f. = 4, *p* = 0.103), followed by species identity (L-ratio = 6.955, d.f. = 4, *p* = 0.138) and environmental condition (L-ratio = 5.952, d.f. = 4, *p* = 0.203). In aquaria containing infauna from station B13 (*A. crenata* and *C. crispatus*), irrespective of species identity, and for *A. crenata* in station B16, the effect size of [NO_2_-N] was not affected by environmental condition (Fig. 3d–e). For station B16, however, the effect size of [NO_2_-N] in aquaria containing *C. crispatus* decreased under future environmental conditions. Similarly, the effect size for nitrate ([NO_3_-N]) in the presence of *A. crenata* or *C. crispatus* was dependent on an environmental condition × location × species identity interaction (F_[1,16]_ = 3.057, *p* = 0.09), with species identity the most influential independent variable (*ω*^2^ = 0.281), followed by location (*ω*^2^ = 0.207) and environmental condition (*ω*^2^ = 0.136). Notably, environmental condition had no effect on the activities of *A. crenata* and *C. crispatus* at station B13 but did influence the behaviour of *C. crispatus* at station B16 (Fig. 3h–i). In contrast, for aquaria with *C. hyperborea,* we find no influence of environmental condition on the effect size of [NO_2_-N] ([F_[1.4]_ = 1.324, *p* = 0.314; Fig. 3f), but the effect size of [NO_3_-N] increased under future conditions (F_1.4_ = 60.821, *p* < 0.01; Fig. 3j). For *L. elliptica* and *A. eightsi*, the effect size of [NO_3_-N] was dependent on the independent effect of environmental condition (L-ratio = 9.720, d.f. = 1, *p* < 0.01; Fig. 3k), with an increased effect size under future conditions for both species.

The effect size for phosphate ([PO_4_-P]) was not dependent on any of our explanatory variables (intercept only model; Fig. 3l) for *A. crenata* and *C. crispatus*. However, we found independent effects of environmental condition on [PO_4_-P] for *C. hyperborea* (L-ratio = 3.123, d.f. = 1, *p* = 0.078; Fig. 3m) and independent effects of environmental condition (L-ratio = 7.865, d.f. = 1, *p* < 0.01) and species identity (L-ratio = 4.662, d.f. = 1, *p* < 0.05) on [PO_4_-P] for *A. eightsi* and *L. elliptica* (Fig. 3n). Intra-specific variability (CV) in the effect size for [PO_4_-P] decreased under future conditions for *A. eightsi* (ambient, 69.7%; future, 50.6%) and *C. hyperborea* (ambient, 68.6%; future, 49.7%), but increased for *L. elliptica* (ambient, 11.7%; future, 47.6%).

## Discussion

Our findings demonstrate that conditions representative of anticipated near-future climate change can lead to fundamental shifts in functionally important aspects of sediment-dwelling invertebrate behaviour. These effects can be substantive; here we observed a doubling of burial rate, deepening of particle mixing and a change in the magnitude and direction of biogeochemical dynamics that are sufficient to change the functional role of a species *(A. eightsi*^36^). This observation is important, because alterations in individual functional capacity that are distinct from functional shifts caused by changes in community composition and/or novel environmental conditions are common^3,61^, and likely result from changes in the strength and nature of a portfolio of sublethal responses, including species interactions^62,63^, compensatory mechanisms^41,42^ and/or other subtle phenotypic responses^54,64^. Changes in macronutrient cycling under climate forcing is not trivial to detect^65^, however, and may be masked by the pH buffering effects of [CO_2_] driven alkalinity changes^66^ on microbial mediated pathways of nutrient recycling.

The behavioural changes with warming and acidification observed here may be even more important ecologically in polar regions than they would be at lower latitudes. Seasonality results in many species entering low energy and activity states similar to aestivation in winter that can last several months^67,68^; in this study, as in *L. elliptica*^69^, although juvenile *A. eightsi* growth is known to be similar across summer and winter^70^. Therefore, in the presence of species that respond to seasonal cues, greater levels of species activity, leading to greater microbial and nutrient remobilisation from sediments^32,33^, may occur for longer in polar regions as the summer season extends under climate change^71^. If widespread, it follows that there will be positive ramifications for phytoplankton productivity over the long term^1,3^. Although this is not the only mechanism underpinning nutrient provision for productivity, we speculate that outcomes associated with benthic responses to climate change could include changes in the phenology of the initiation of productivity and early intensity of phytoplankton growth^72^, with downstream impacts for primary and secondary consumers.

Whilst the effects of a near-future climate in our experiments were comparatively weaker than the effects of species identity and location, consistent with theoretical expectations^73,74^, we did note a reduction in intra-specific variation that reflected changes in environmental context and location^37^. This can be very important for maintaining populations^75^, enabling adaptation to changing environmental conditions^76^ and stability in ecosystem functioning^77^. However, whilst sublethal responses may enable species to persist in, or for longer, under novel circumstances, other phenotypic costs may constrain or inhibit an individual’s ability to adjust further^78,79^. Hence, reductions in intra-specific variation may serve as an early warning for impending ecological transitions associated with progressive forcing and potentially inform more timely management actions, reinforcing the need for continual monitoring of faunal activity and the ecological constraints that modify functionally important aspects of species behaviour^80^.

The variation in intra-specific behaviour observed here under enhanced warming and [CO_2_] is consistent with other behavioural studies^81^, physiological responses in polar benthic species^21^ and incorporates regional contextualisation^13^. Whilst our study was not explicitly designed to examine species range shifts or gradients of environmental change, an important feature of our sampling design was that our locations were positioned to the north and south of the oceanographic polar front, contrasting in benthic biogeography^82^, bioturbation activity and functioning^3^. Hence, we were able to show that individuals predisposed to a wider inter-annual thermal range exhibit a more reserved behavioural response to change than those inhabiting a narrower thermal range^83^. Thus, plasticity in response mirrors the level of local environmental fluctuation^84^. Whilst spatial associations between environmental temperature range and physiological thermal tolerances are not atypical in ectothermic species^13,85,86^, this does mean that high latitude populations may be at greater risk of local extinction over the long term. As thermal tolerance narrows with decreasing seasonality in temperature towards the poles^16,87^, and will likely be further constrained with ocean warming^88^, populations already at or approaching the edge of their thermal limits will most likely have less scope to compensate and adapt to change^89^. Indeed, changes in species composition and abundance are well documented across areas of environmental transition^3^ and show similar patterns of functional change, as observed here. Temperature-driven responses are, however, typically complicated by interactions with other abiotic drivers^74^ and are likely to lead to both amplified and dampened effects in spatially stochastic ecosystems^90^. Yet, previous investigations have predominantly focused on spatial distributions of species turnover^64^, functional diversity^91,92^ and redundancy^93^, rather than characterising intraspecific variability of species-environment interactions. The latter can be a more important driver of the short-term functional response of communities than changes in species composition, dominance, or richness^94,95^. For example, the shallower burrowing activity of invertebrates held under more acidified conditions^96^ allows species to evade the physiological effects of decreasing pH, but simultaneous burrowing and ventilatory^40^ responses to warming to maintain environmental continuity may negate the need for such avoidance behaviour^97^. We observed similar changes across multiple aspects of functionally important behaviour that may have led to non-additive effects on net functioning that were not possible to distinguish. Nevertheless, the cumulative effect of such short-term behavioural responses is likely to be decisive for the composition^28^, population dynamics^98^, connectivity^99^ and functioning^100^ of benthic communities that will be moderated by seasonal timing^54^ and local circumstance^13,36^, including interannual variability^3^.

Quantitative information on the functional role of individual species is rare for both polar regions^101^, yet understanding, and accounting for, species responses to climate change is fundamental to improving the likelihood of determining the most realistic ecosystem future^102^. We contend that this task will be frustrated by context-dependent variation in both intra- and inter-specific responses to forcing that are not readily captured using fixed trait modalities^35,103^. Where the overall outcome of species responses remains largely unresolved, reductions in the variation of conspecific responses^95,104^ may form a viable alternative for some predictive models. Furthermore, our findings lend support to the view that location-dependent variation in behavioural responses can be attributed to localised thermal plasticity driven by exposure to divergent temperature seasonality trends^8,84,105^. Inter- and intra-specific variations in vulnerability, effect-and-response traits^79^ and interactions between species^106,107^ can facilitate functional redundancy and/or post-change compensations^42,43^. A mechanistic approach that explicitly tests suspected abiotic and biotic signals is necessary for establishing patterns of response^108^ across multiple levels of biological organisation^109,110^, enabling the generation of more robust projections of the most likely functional consequences of change.

## Material and methods

### Fauna and sediment collection

We obtained individuals of the bivalve *Astarte crenata*, sea star *Ctenodiscus crispatus* and polychaete *Cistenides hyperborea* from replicate SMBA (Scottish Marine Biological Association, 50 × 50 cm) box cores, and 15 min Agassiz trawls in the Barents Sea (stations B13, 74.3° N, 30.0° E; B16, 80.3° N, 30.0° E, 263–375 m depth; JCR18006, *RSS James Clark Ross,* Supplementary Fig. S1a, Table S1) in July 2019. Individuals of the protobranch *Aequiyoldia eightsi* and bivalve *Laternula elliptica* were collected by SCUBA divers at Rothera Point, Adelaide Island, West Antarctic Peninsula (67.3° S, 68.1° W, 10–20 m depth, Supplementary Fig. S1b) in March–April 2019. We obtained surficial sediment (< 5 cm depth) from SMBA box cores at the Barents Sea stations B13, B14 and B16 (Supplementary Table S1) for the Arctic species, and from the intertidal mud flats of the Hamble, UK (50.9° N, 1.3° W) for the Antarctic species. Each sediment was sieved (500 µm) within a seawater bath to retain the fine fraction and to remove macrofauna and debris. Sediment particle size (Supplementary Fig. S2) was determined using a Malvern Mastersizer 2000 He–Ne LASER diffraction sizer. Mean particle size, sorting, skewness and kurtosis were quantified using GRADISTAT^111^. Loss on ignition was used to determine sediment organic matter content (%).

### Experimental design and set-up

Sediment (mean ± s.e., n = 38: particle size = 60.30 ± 3.91 µm, organic matter content = 5.502 ± 0.212%; Supplementary Table S2) and species were distributed across 42 clear acrylic aquaria (internal LWH: 12 × 12 × 33 cm, 3 replicates treatment^−1^: species × location × climate scenario; Supplementary Table  S1), designed to accommodate representative field densities (Arctic species, 2 ind. aquarium^−1^; Antarctic species, 1 ind. Aquarium^−1^; (^112^; Supplementary Table S4) and the size and burrowing requirements of each species (sediment depth: *A. crenata, C. crispatus & C. hyperborea,* 16 cm; *A. eightsi*, 12 cm; *L. elliptica*, 19 cm^113,114^). Aquaria were randomly placed within one of two insulated seawater reservoirs (^3^, Supplementary Fig. S3) in the *Biodiversity and Ecosystem Futures Facility*, University of Southampton (UK). All aquaria were filled with seawater (salinity 33, 10 µm sand filtered, UV sterilized) to ~ 12 cm above the sediment–water interface and maintained in the dark. After a transitionary period to aquarium conditions (21 days, 09–29/09/2019), fauna was exposed to ambient (1 ± 0.5 °C, ~ 400 ppm atmospheric [CO_2_]) or indicative near-future (2.5 ± 0.5 °C, ~ 550 ppm atmospheric [CO_2_]) environmental conditions. Water temperature and atmospheric [CO_2_] were increased from ambient to treatment levels in 0.5 °C and 50 ppm increments every 7 days (21 days, 29/09/2019–20/10/2019) to minimise adverse physiological responses^115^. During both the transitionary and experimental period (92 days, 21/10/2019–21/01/2020), species were fed ad libitum; *C. crispatus* and *C. hyperborea* with commercially available fish food (Aquarian Tropical Flake; 0.03 g week^−1^), and *A. crenata*, *A. eightsi* and *L. elliptica* with precultured phytoplankton (15 ml, 3 × week^−1^, 33:33:33 mix: *Isochrysis* sp., *Tetraselmis* sp., *and Phaeodactylum* sp.). This period of time was sufficient for the establishment of microniche formation^116^ and vertical biogeochemical gradients indicated by colour change^117^ to form in the sediment. Partial seawater exchanges (weekly, 50% volume) prevented accumulation of excess food and nutrients. Measurements in behaviour, ecosystem process and functioning were taken at the end of the experimental period.

### Seawater carbonate chemistry, temperature, and salinity

Atmospheric [CO_2_] (Supplementary Fig. S4) was controlled using a custom-made CO_2_-air mixing system which continually maintained and monitored [CO_2_] in the air mixture supplied to each individual experimental core using infrared analysers (LI-COR LI-840A)^54^. This approach facilitates natural variability within the carbonate system^118^.Temperature, pH (NBS scale, Mettler-Toledo InLab Expert Pro temperature-pH combination electrode; weekly three-point calibration using technical buffer solutions pH 4.01, 7.00, 9.21, Mettler-Toldedo), and salinity (WTW™ TetraCon™ 325 Standard conductivity electrode; weekly calibration using conductivity standard solution 12.88mS, Mettler-Toldedo) were measured weekly and total alkalinity (A_T_, Apollo SciTech Titrator AS-ALK2) was measured in weeks 2, 6 and 11 in each experimental core. A_T_ analysis followed standard HCl titration protocols of the *Carbonate Facility*, University of Southampton. DIC, [pCO_2_], [Ω_calcite_], [Ω_aragonite_], [NCO_3_] and [CO_3_] were calculated (*CO2calc* carbon calculator, v 4.0.9) (^119^; Supplementary Figs. S5 and S6).

### Behavioural response of individuals

Behaviour of *C. crispatus, C. hyperborea* and *A. eightsi* were quantified using measurements of movement and burial behaviour at the sediment surface. Individuals (morphology, ± 0.01 mm; blotted wet weight, ± 0.001 g, Supplementary Table S5) were placed in separate treatment-acclimatised viewing trays containing sediment (depth 5 cm) overlain with sea water (depth 3 cm) and viewed (≤ 60 min) with a benchtop video camera (Logitech C920 HD Pro, 1080p; Supplementary Fig. S7). The time taken to initiate movement (response time, s) and to complete burial (burial time, s) was recorded (3 frame s^−1^, SkyStudioPro) and analysed frame-by-frame (VLC Media Player). We incorporated biomass as a random factor in the statistical analysis to account for any intra-specific variation in size.

### Effects on ecosystem process and functioning

Sediment particle reworking activity of all five species was determined from the redistribution of fluorescent particulate luminophore tracers (30 g aquarium^−1^, 125–250 μm diameter, 12 days 09/01/2020–21/01/2020^120^). All four aquarium sides were imaged under UV light (Canon EOS 400D, 3888 × 2592 pixels, effective resolution 74 × 74 μm pixel^−1^), stitched together (Adobe Photoshop CC 2019; Supplementary Figs. S8–S12), and the distribution of luminophores was analysed using ImageJ (version 1.46r^120^). From these profile data (Supplementary Fig. S13), we calculated the mean (^f-SPI^L_mean_, time dependent indication of mixing), median (^f-SPI^L_med_, typical short-term depth of mixing) and maximum (^f-SPI^L_max_, maximum extent of mixing) mixed depth of particle redistribution. Given the shape of the vertical distribution of luminophores (non-continuous), ^f-SPI^L_mean_ was an unsuitable descriptor of the distribution profile and not considered for statistical analysis. The rugosity of the sediment–water interface (upper–lower limit = surface boundary roughness, SBR) provides an indication of surficial activity.

Ventilation behaviour^101^ of all five species was estimated from absolute changes in the concentration of sodium bromide [NaBr]^54^. Dissolved [NaBr] was standardised across all aquaria (mean starting concentration = 1353.816 ± 317.264 mg L^−1^) and [NaBr] was determined using a Tecator flow injection auto-analyser (FIA Star 5010 series). Negative values of [NaBr] (∆[Br^−^] mg L^−1^) over an 8-h period indicate increased infaunal ventilatory activity.

As faunal activity mediates nutrient concentrations, we determined water column [NH_4_-N], [NO_3_-N], [NO_2_-N] and [PO_4_-P] (µmol L^−1^, ~ 10 ml, filtered 0.45 μm NALGENE nylon matrix) for all five species once a month (Supplementary Fig. S14) using a QuAAtro 39 auto-analyser (SEAL Analytical) as a measure of ecosystem functioning. As nutrient concentrations will also reflect differences in the volume of sediment between species treatments, we calculated the log response ratio (lnRR = ln[conc_before_/conc_after_]^121^), an effect size that quantifies proportionate change. As patterns of [NO_x_-N] are reciprocal to those of [NH_4_-N] but indicate beneficial biogeochemical processes (e.g. denitrification), lnRR values for [NO_2_-N] and [NO_3_-N] were multiplied by −1 to align the direction of ecosystem functioning.

### Statistical analysis

Analysis of Variance (ANOVA) models were developed for each dependent variable (movement and burial behaviour: response time, burial time; ecosystem process: SBR, ^f-SPI^L_median,_^f-SPI^L_max_, ∆[Br^−^]; ecosystem functioning: [NH_4_-N], [NO_3_-N], [NO_2_-N], [PO_4_-P]). For *A. crenata* and *C. crispatus*, we determined the effects of the independent variables; environmental condition (2 levels: ambient, future), location (2 levels: stations B13 and B16; Supplementary Fig. S1a), species identity (2 levels), and their interactions, whilst for *A. eightsi* and *L. elliptica*, we determined the effects, alone and in combination, of the independent variables environmental condition (2 levels) and species identity (2 levels). As *C. hyperborea* was found at a single station, we determined only the effects of environmental condition (2 levels). Intra-specific variability within treatment levels was determined using the coefficient of variation.

Model assumptions were visually assessed using standardised residuals vs fitted values plots, Q-Q plots, and Cook’s distance^122^. Where there was a violation of homogeneity of variance, we used a *varIdent* variance–covariance structure and generalised least-squares (GLS) estimation^123,124^ to allow residual spread to vary amongst groups. We determined the optimal fixed-effects structure using backward selection informed by Akaike Information Criteria (AIC) and inspection of model residual patterns. For the GLS analysis, we determined the optimal variance–covariance structure using restricted maximum-likelihood (REML) estimation by comparing the initial ANOVA model without variance structure to equivalent GLS models incorporating specific variance terms. These models were compared against the initial ANOVA model using AIC informed by visualisation of model residuals. We determined the optimal fixed structure of the most suitable model by applying backward selection using the likelihood ratio test with maximum-likelihood (ML) estimation^122,124^. For ANOVA models with interactions, we calculated the effect size (*ω*^2^^125^) of each independent variable in R^126^ using the *effectsize* package^127^. For GLS models with interactions, we determined the relative importance of each independent variable by comparing the minimal adequate model with a model with the independent variable of interest, and all its interactions, removed using likelihood ratio (L-ratio) in the *nlme* package^123^. Details of initial and minimal adequate models (Model S1 to S29) are provided in electronic supplementary material.

### Supplementary Information

Supplementary Information.

## Data Availability

All data associated with this analysis are available at the Polar Data Centre (https://www.bas.ac.uk/data/uk-pdc/; 10.5285/7adc7b14-abae-4ab9-b60b-b9b6e0e9f320; Data records S1). Extended data items, including the “minimum datasets” that are necessary to interpret, verify and extend the research in the article, can be found in the electronic supplementary material.
