# Supplementary material for: Ocean warming and acidification adjust inter- and intra-specific variability in the functional trait expression of polar invertebrates
Source: Sci Rep. 2024 Jul 1;14:14985. doi: 10.1038/s41598-024-65808-5 (PMC11217501; doi:10.1038/s41598-024-65808-5)
Supplement: Supplementary file 1 — Supplementary Information. [file 41598_2024_65808_MOESM1_ESM.pdf]

Electronic supplementary material for

Williams TJ, Reed AJ, Peck, LS, Godbold JA, Solan M. Ocean warming and acidification adjust inter- and intra-specific variability in the functional trait expression of polar invertebrates. *Sci. Rep.*

Data records S1.

Data records are available *via* an unrestricted repository hosted by the Discovery Metadata System (https://www.bas.ac.uk/project/dms/), a data catalogue hosted by The UK Polar Data Centre (UK PDC, https://www.bas.ac.uk/data/uk-pdc/).

The following data records were used in this contribution:

Experimental measurements

Williams, TJ, Reed, AJ, Peck, LS, Godbold, JA, & Solan, M. 2023. Measurements for a 92-day climate manipulation experiment on replicate macrofaunal mesocosms collected on the cruise JR18006 in the Western Barents Sea and at Rothera research station in 2019 (Version 1.0) [Data set]. NERC EDS UK Polar Data Centre. https://doi.org/10.5285/7ADC7B14-ABAE-4AB9-B60B-B9B6E0E9F320

The cruise report (RRS James Clarke Ross, JR18006) is available here:

https://www.bodc.ac.uk/resources/inventories/cruise_inventory/reports/jr18006.pdf

Figure S1: The location of (*a*) the stations B13 and B16 in the Barents Sea relative to the generally accepted position of the oceanographic (Loeng, 1991, grey line) and benthic (Jørgensen *et al.* 2014, dashed black line) polar front, and (*b*) the Rothera Point station, western Antarctica.

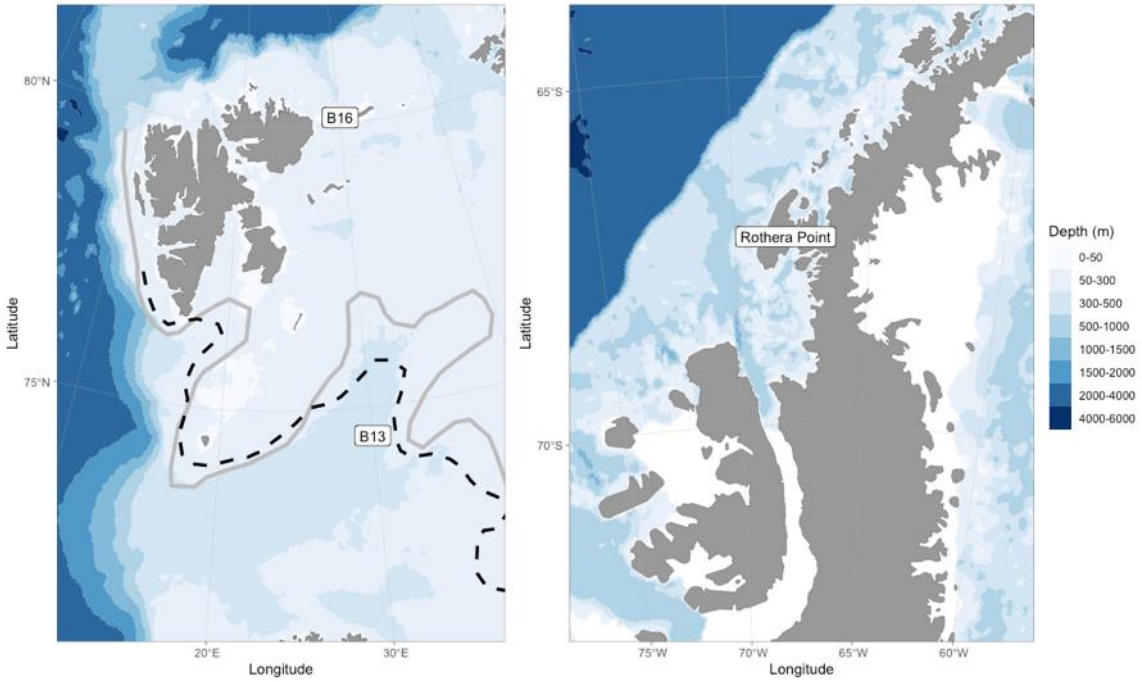

Table S1 (next two pages): Summary of the cruise event number from the ship log, date, timing, geographical position and water column depth for each (a) box core and (b) trawl obtained for the collection of sediment (S) and/or sediment-dwelling macrofaunal invertebrates (F) obtained during research cruise JR18006, RRS James Clark Ross (Barnes et al. 2018).

Table S1(a)

Station	Event	Date	Lat (˚N)	Long (˚E)	Time (UTC)	Depth (m)
B13	$32_{S,F}$	08/07/2019	74.46607	30.11835	18:14:45	354.59
B13	$33_{S,F}$	08/07/2019	74.46613	30.11831	18:52:08	354.21
B13	$34_{S,F}$	08/07/2019	74.46614	30.1184	19:23:06	354.19
B13	$35_{S,F}$	08/07/2019	74.46621	30.1184	20:02:01	357.10
B13	$36_{S,F}$	08/07/2019	74.46618	30.11864	20:33:04	354.41
B13	$37_{S,F}$	08/07/2019	74.46623	30.11868	21:05:04	354.38
B13	$38_{S,F}$	08/07/2019	74.46619	30.119	21:50:21	353.99
B13	$39_{S,F}$	08/07/2019	74.46626	30.11901	22:29:06	254.18
B13	$40_{S,F}$	08/07/2019	74.46625	30.1191	23:05:50	353.91
B13	$41_{S,F}$	08/07/2019	74.46627	30.11932	23:36:46	353.82
B13	$42_{S,F}$	09/07/2019	74.46632	30.1194	00:18:57	353.97
B13	$43_{S,F}$	09/07/2019	74.46632	30.11948	00:53:47	353.50
B13	$44_{S,F}$	09/07/2019	74.46631	30.11958	01:30:14	354.32
B14	105_{S}	13/07/2019	76.55291	30.61992	09:00:16	281.47
B14	106_{S}	13/07/2019	76.55282	30.61963	09:38:10	281.41
B16	$161_{S,F}$	17/07/2019	80.08478	30.15126	06:00:25	263.00
B16	$162_{S,F}$	17/07/2019	80.08561	30.14997	06:28:22	264.00
B16	$163_{S,F}$	17/07/2019	80.08785	30.1499	07:04:36	264.00

Table S1(b)

Station	Event	Date	Latitude (˚N)			Longitude (˚E)			Time (HH:MM, UTC)			Trawl time (mins)	Depth(m)
			On bottom	Trawl Start	Left bottom	On bottom	Trawl Start	Left bottom	On bottom	Trawl Start	Left bottom		
B13	53_F	09/07/2019	74.4972	74.49767	74.49857	30.0744	30.07926	30.08872	10:39	10:49	11:09	00:19	361.86
B13	54_F	09/07/2019	74.50071	74.50109	74.50149	30.11158	30.11768	30.12638	12:24	12:36	12:53	00:17	351.78
B13	171_F	24/07/2019	74.49889	74.4973	74.4963	29.99775	29.98403	29.97519	16:43	17:12	17:30	00:18	362.15
B13	172_F	24/07/2019	74.49567	74.49566	74.49568	29.9643	29.95472	29.94587	18:11	18:29	18:46	00:17	367.31
B13	173_F	24/07/2019	74.4964	74.49767	74.49861	29.93578	29.92737	29.92129	19:30	19:49	20:02	00:13	371.81
B13	174_F	24/07/2019	74.50068	74.50213	74.50327	29.90772	29.89843	29.89113	21:02	21:22	21:38	00:16	374.83
B13	175_F	24/07/2019	74.50524	74.50657	74.50765	29.87843	29.86988	29.86281	22:25	22:44	23:00	00:15	372.55
B13	176_F	24/07/2019	74.51009	74.5109	74.51229	29.84718	29.84188	29.83291	23:46	00:03	00:20	00:17	372.81
B13	177_F	25/07/2019	74.51637	74.51866	74.52021	29.81048	29.80509	29.80134	01:13	01:33	01:46	00:13	369.16

Figure S2: Cumulative sediment particle size distributions (n = 3) for the sediments used in aquaria containing *Astarte crenata* from (a) station B13 and (b) station B16, *Ctenodiscus crispatus* from (c) station B13 and (d) station B16, (e) *Cistenides hyperborea* from station B13, (f) *Aequiyoldia eightsi* and (g) *Laternula elliptica* analysed following standard protocols at the Department of Geography, University of Cambridge (available at: www.geog.cam.ac.uk/facilities/laboratories/techniques/). Line colour indicates aquaria maintained under ambient (black) versus future (red) climate conditions.

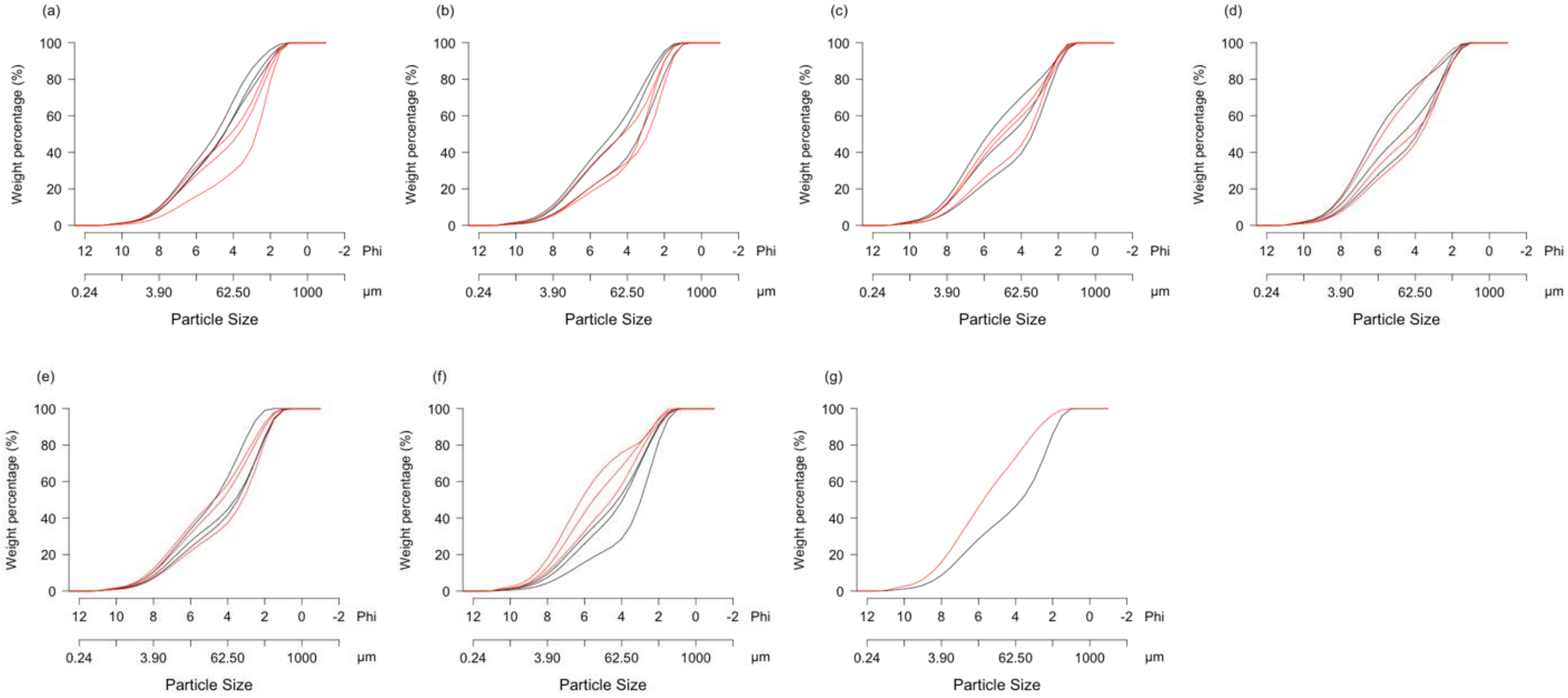

Table S2 (next two pages): Summary of sediment particle size statistics for each replicate aquaria determined from profile distributions of particle size using GRADISTAT (Blott & Pye, 2001). Mean, sorting, skewness, kurtosis, the percentage of sample less than 63 µm and organic matter content (%) are presented for all aquaria maintained in (i) ambient and (ii) future climate conditions. Superscripts provide descriptive terminology as outlined by Blott & Pye (2001). **<u>Mean, $\bar{x}$</u>:** vfs, very fine sand, fs, fine sand; ms, medium silt; cs, coarse silt; vcs, very coarse silt. **<u>Sorting, *σ*</u>:** ps, poorly sorted; vps, very poorly sorted. **<u>Skewness, *Sk*</u>:** sy, symmetrical; vfsk, very fine skewed; fsk, fine skewed; csk, coarse skewed. **<u>Kurtosis, *K*</u>:** mk, mesokurtic; lk, leptokurtic; pk, platykurtic.

Table S2

Species identity	Station	Climate	Mean (x̄, µm)	Sorting (σ, µm)	Skewness (Sk, µm)	Kurtosis (K, µm)	Sample <63 µm (%)	Organic matter content (%)	Sediment type
**i. Ambient**									
Astarte crenata	B13	Ambient	51.86^{vcs}	4.444^{vps}	-0.183^{fsk}	0.855^{pk}	50.180	4.547	Very Coarse Silty Very Fine Sand
Astarte crenata	B13	Ambient	39.50^{vcs}	4.233^{vps}	-0.150^{fsk}	0.878^{pk}	58.250	4.569	Very Fine Sandy Very Coarse Silt
Astarte crenata	B13	Ambient	54.08^{vcs}	4.762^{vps}	-0.142^{fsk}	0.828^{pk}	50.480	4.625	Very Fine Sandy Very Coarse Silt
Astarte crenata	B16	Ambient	91.19^{vcs}	4.644^{vps}	-0.423^{vfsk}	0.910^{mk}	31.940	4.911	Coarse Silty Fine Sand
Astarte crenata	B16	Ambient	43.40^{vfs}	4.716^{vps}	-0.164^{fsk}	0.781^{pk}	53.990	4.957	Fine Sandy Medium Silt
Astarte crenata	B16	Ambient	53.49^{vcs}	4.642^{vps}	-0.267^{fsk}	0.772^{pk}	47.590	4.591	Medium Silty Fine Sand
Ctenodiscus crispatus	B13	Ambient	32.58^{vcs}	5.424^{vps}	0.134^{csk}	0.794^{pk}	65.120	5.004	Medium Sandy Medium Silt
Ctenodiscus crispatus	B13	Ambient	81.62^{cs}	4.588^{vps}	-0.460^{csk}	0.863^{mk}	34.260	4.910	Medium Silty Fine Sand
Ctenodiscus crispatus	B13	Ambient	48.03^{vcs}	5.163^{vps}	-0.207^{fsk}	0.719^{pk}	50.670	5.100	Fine Sandy Medium Silt
Ctenodiscus crispatus	B16	Ambient	63.32^{vcs}	4.620^{vps}	-0.378^{fsk}	0.789^{pk}	41.620	5.353	Coarse Silty Fine Sand
Ctenodiscus crispatus	B16	Ambient	27.01^{vfs}	4.983^{vps}	0.193^{vfsk}	0.901^{pk}	71.450	5.223	Medium Sandy Medium Silt
Ctenodiscus crispatus	B16	Ambient	47.69^{vfs}	5.388^{vps}	-0.114^{vfsk}	0.728^{pk}	52.550	5.125	Fine Sandy Medium Silt
Cistenides hyperborea	B13	Ambient	80.77^{vfs}	4.850^{vps}	-0.422^{vfsk}	0.818^{pk}	36.410	4.322	Medium Silty Medium Sand
Cistenides hyperborea	B13	Ambient	73.97^{vfs}	5.209^{vps}	-0.380^{vfsk}	0.760^{pk}	39.690	4.876	Medium Silty Medium Sand
Cistenides hyperborea	B13	Ambient	42.26^{vcs}	4.280^{vps}	-0.246^{fsk}	0.796^{pk}	53.590	4.853	Fine Sandy Very Coarse Silt
Aequiyoldia eightsi	Rothera	Ambient	57.41^{vcs}	4.900^{vps}	-0.267^{fsk}	0.780^{pk}	46.180	4.898	Medium Silty Fine Sand
Aequiyoldia eightsi	Rothera	Ambient	115.6^{vfs}	3.963^{ps}	-0.500^{vfsk}	1.188^{lk}	24.490	4.392	Coarse Silty Fine Sand
Aequiyoldia eightsi	Rothera	Ambient	66.30^{vfs}	4.574^{vps}	-0.311^{vfsk}	0.837^{pk}	41.720	4.750	Very Coarse Silty Fine Sand
Laternula elliptica	Rothera	Ambient	70.28^{vfs}	5.090^{vps}	-0.375^{vfsk}	0.732^{pk}	41.660	4.616	Medium Silty Medium Sand
**ii. Future**									
Astarte crenata	B13	Future	122.0^{vfs}	4.024^{vps}	-0.575^{vfsk}	1.067^{mk}	25.630	4.668	Very Coarse Silty Medium Sand
Astarte crenata	B13	Future	58.32^{vcs}	5.055^{vps}	-0.271^{fsk}	0.728^{pk}	46.440	4.961	Medium Silty Fine Sand
Astarte crenata	B13	Future	70.10^{vfs}	5.019^{vps}	-0.372^{vfsk}	0.755^{pk}	41.010	4.962	Medium Silty Medium Sand

Astarte crenata	B16	Future	104.9^{vfs}	4.708^{vps}	-0.536^{vfsk}	0.844^{pk}	31.060	5.184	Medium Silty Medium Sand
Astarte crenata	B16	Future	57.16^{vcs}	5.095^{vps}	-0.247^{fk}	0.722^{pk}	47.620	5.295	Medium Silty Fine Sand
Astarte crenata	B16	Future	92.39^{vfs}	3.997^{ps}	-0.491^{vfsk}	1.096^{mk}	28.100	4.448	Very Coarse Silty Fine Sand
Ctenodiscus crispatus	B13	Future	40.20^{vcs}	5.263^{vps}	0.014^{sy}	0.722^{pk}	57.550	4.671	Fine Sandy Medium Silt
Ctenodiscus crispatus	B13	Future	69.43^{vfs}	4.487^{vps}	-0.448^{vfsk}	0.790^{pk}	38.460	4.600	Medium Silty Fine Sand
Ctenodiscus crispatus	B13	Future	45.91^{vcs}	5.394^{vps}	-0.036^{sy}	0.716^{pk}	54.690	5.098	Medium Sandy Medium Silt
Ctenodiscus crispatus	B16	Future	71.50^{vfs}	4.614^{vps}	-0.390^{vfsk}	0.809^{pk}	38.790	5.147	Medium Silty Fine Sand
Ctenodiscus crispatus	B16	Future	59.65^{vcs}	5.063^{vps}	-0.279^{fsk}	0.717^{pk}	46.430	4.963	Medium Silty Fine Sand
Ctenodiscus crispatus	B16	Future	29.09^{cs}	4.698^{vps}	0.074^{sy}	0.831^{pk}	67.570	7.282	Very Fine Sandy Medium Silt
Cistenides hyperborea	B13	Future	54.81^{vcs}	5.097^{vps}	-0.238^{fsk}	0.777^{pk}	47.800	7.210	Coarse Silty Fine Sand
Cistenides hyperborea	B13	Future	45.47^{vcs}	5.192^{vps}	-0.155^{fsk}	0.758^{pk}	52.480	7.985	Fine Sandy Medium Silt
Cistenides hyperborea	B13	Future	91.16^{vfs}	4.791^{vps}	-0.478^{vfsk}	0.847^{pk}	32.820	8.059	Coarse Silty Medium Sand
Aequiyoldia eightsi	Rothera	Future	34.43^{vcs}	4.981^{vps}	0.061^{sy}	0.779^{pk}	62.490	8.481	Fine Sandy Medium Silt
Aequiyoldia eightsi	Rothera	Future	27.23^{cs}	5.448^{vps}	0.210^{csk}	0.865^{pk}	72.010	8.275	Medium Sandy Medium Silt
Aequiyoldia eightsi	Rothera	Future	48.88^{vcs}	4.922^{vps}	-0.200^{fsk}	0.818^{pk}	50.670	8.123	Fine Sandy Coarse Silt
Laternula elliptica	Rothera	Future	28.32^{cs}	4.812^{vps}	0.029^{sy}	0.834^{pk}	66.860	8.026	Very Fine Sandy Medium Silt

Table S3: Summary of experimental design for investigating the effects of enhanced temperature and atmospheric [CO_2] on species-specific behaviour and associated contributions to ecosystem process and functioning.

Species	Polar Region	Station	total $n_{aquaria}$	Climate regimes	
Astarte crenata	Arctic	B13, B16	12	1°C, $400_{[CO2]}$ ppm	2.5°C, $550_{[CO2]}$ ppm
Ctenodiscus crispatus	Arctic	B13, B16	12	1°C, $400_{[CO2]}$ ppm	2.5°C, $550_{[CO2]}$ ppm
Cistenides hyperborea	Arctic	B13	6	1°C, $400_{[CO2]}$ ppm	2.5°C, $550_{[CO2]}$ ppm
Aequiyoldia eightsi	Antarctic	Rothera	6	1°C, $400_{[CO2]}$ ppm	2.5°C, $550_{[CO2]}$ ppm
Laternula elliptica	Antarctic	Rothera	6	1°C, $400_{[CO2]}$ ppm	2.5°C, $550_{[CO2]}$ ppm

Table S4 (next three pages): Morphological measurements of (a) *Astarte crenata* (b) *Ctenodiscus crispatus* (c) *Cistenides hyperborea* (d) *Aequiyoldia eightsi* and (e) *Laternula elliptica* listed by each climate treatment group (ambient vs future) .

Table S4(a)

Climate	Station	Shell Length (mm)	Shell Height (mm)	Shell Width (mm)
Ambient	B13	25.87	21.97	11.81
Ambient	B13	20.09	17.58	9.15
Ambient	B13	24.72	19.91	10.70
Ambient	B13	22.95	18.43	9.05
Ambient	B13	27.58	24.57	11.18
Ambient	B13	25.07	20.28	11.18
Ambient	B16	28.12	24.78	12.65
Ambient	B16	28.29	21.55	11.69
Ambient	B16	23.97	19.52	11.75
Ambient	B16	22.97	18.98	11.39
Ambient	B16	28.76	23.90	12.69
Ambient	B16	22.97	18.98	11.39
Future	B13	24.28	20.51	10.48
Future	B13	22.37	18.65	10.28
Future	B13	19.49	16.16	7.48
Future	B13	21.08	18.13	8.88
Future	B13	18.14	16.51	8.89
Future	B13	17.68	14.61	7.38
Future	B16	29.63	24.77	12.44
Future	B16	30.16	25.37	12.55
Future	B16	29.19	24.71	12.97
Future	B16	28.40	22.95	11.71
Future	B16	27.14	22.26	13.21
Future	B16	26.04	20.47	12.37

Table S4(b)

Climate	Station	Arm length (mm)	Pit length (mm)
Ambient	B13	17.53	10.38
Ambient	B13	13.13	8.41
Ambient	B13	18.17	11.11
Ambient	B13	16.98	10.48
Ambient	B13	13.90	10.88
Ambient	B13	17.25	10.13
Ambient	B16	23.33	16.13
Ambient	B16	17.78	10.10
Ambient	B16	10.64	8.32
Ambient	B16	14.79	7.33
Ambient	B16	18.78	10.88
Ambient	B16	11.52	6.98
Future	B13	16.75	11.08
Future	B13	19.49	12.38
Future	B13	17.05	11.05
Future	B13	15.32	9.44
Future	B13	11.74	9.27
Future	B13	14.40	8.66
Future	B16	16.83	10.88
Future	B16	17.13	9.07
Future	B16	17.08	11.65
Future	B16	15.99	10.06
Future	B16	15.12	9.45
Future	B16	11.70	7.99

Table S4(c)

Climate	Station	Cone length (mm)	Anterior aperture (mm)
Ambient	B13	60.68	8.08
Ambient	B13	57.09	7.14
Ambient	B13	57.98	8.03
Ambient	B13	67.38	8.69
Ambient	B13	66.07	6.98
Ambient	B13	35.78	6.74
Future	B13	55.18	7.36
Future	B13	56.23	7.58
Future	B13	40.88	5.95
Future	B13	61.38	7.88
Future	B13	59.16	7.78
Future	B13	48.74	6.88

Table S4(d)

Climate	Shell Length (mm)	Shell Height (mm)	Shell Width (mm)
Ambient	22.42	14.11	6.41
Ambient	16.36	11.00	3.99
Ambient	19.11	11.75	5.90
Future	19.28	12.56	5.80
Future	24.12	15.01	7.55
Future	20.90	13.43	6.66

Table S4(e)

Climate	Shell Length (mm)	Shell Height (mm)	Shell Width (mm)
Ambient	64.71	44.88	34.20
Ambient	61.97	41.85	27.63
Ambient	66.98	49.33	34.09
Future	50.82	38.41	26.53
Future	48.38	36.52	27.48
Future	72.63	50.61	37.16

Figure S3: System of (a) interconnected insulated fibreglass seawater baths (lids removed, LWH: 1.2 x 1.2 x 0.8m) used to house the aquaria (following Table S1), with temperature controlled by a chiller (located top left of panel (a)). Aquaria were randomly allocated to water baths within a climate treatment, randomly positioned within each water bath, and (b) continually aerated by bubbling into the water column through a glass pipette linked to a controllable air supply (grey ducting). The green coloration in each aquarium is the luminophore particulate tracers used to track infaunal particle mixing. Water bath temperatures were controlled (± 1˚C). Water buckets pictured were used for routine partial water exchanges and pre-chilled to match the temperature of each climate treatment.

Figure S3(a)

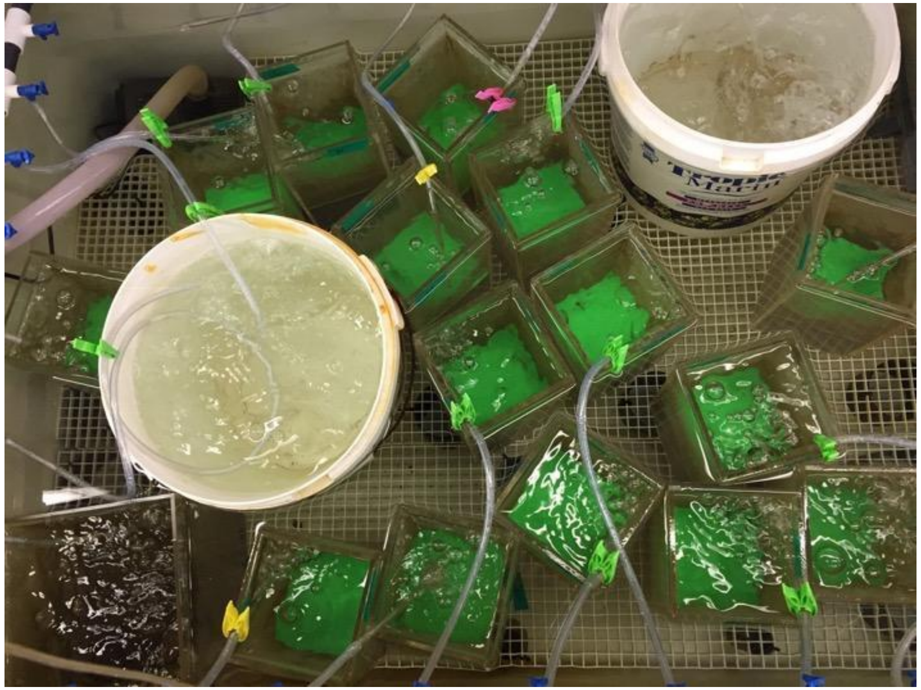

Figure S3(b)

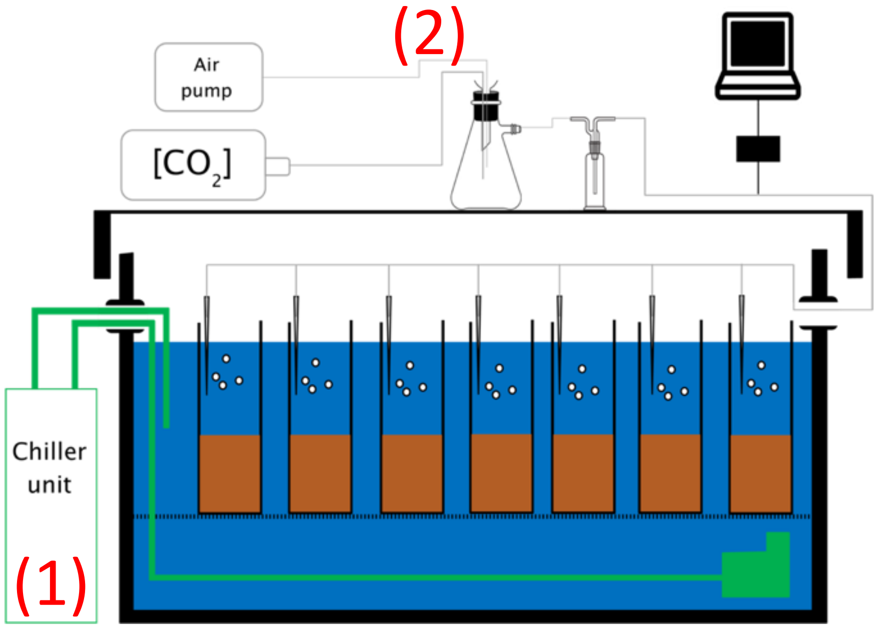

Figure S4: Recorded mean daily atmospheric [CO_2] (ppm) that was bubbled into all aquaria maintained under ambient (blue dot-dash line) and future (red line) environmental conditions (92 days; 21st October 2019 to 21st January 2020). The reduction in [CO_2] in the future treatment at day 54-57 corresponds to a system failure. Other large departures in [CO_2] correspond to periods of maintenance where personnel were in the experimental room for long periods of time. [CO_2] concentrations were measured and monitored continuously with an infrared gas analyser (Licor LI-840A). Filled area around the trend is representative of 1 standard deviation.

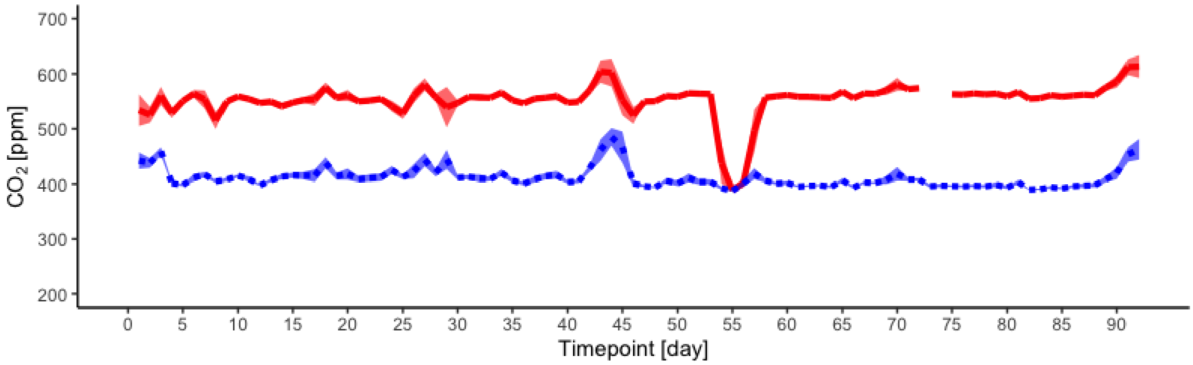

Figure S5: Seawater carbonate chemistry measurements in aquaria containing *Astarte crenata* (circles), *Ctenodiscus crispatus* (squares), *Cistenides hyperborea* (triangle), *Aequiyoldia eightsi* (diamond) and *Laternula elliptica* (upside down triangles) maintained under ambient (blue) and future (red) climate conditions. Weekly temperature (°C), Salinity, pH_{NBS} and monthly total alkalinity (A_T, µmol $kgSW^{-1}$) were measured directly from each aquarium and were used to calculate dissolved organic carbon (DIC, µmol $kgSW^{-1}$), pCO_2^{SW} (µAtm), saturation states for calcite ($\Omega_{Calcite}$) and aragonite ($\Omega_{Aragonite}$), bicarbonate (HCO_3, µmol $kgSW^{-1}$) and carbonate (CO_3, µmol $kgSW_{-1}$) using *CO2calc* (Robbins et al., 2010).

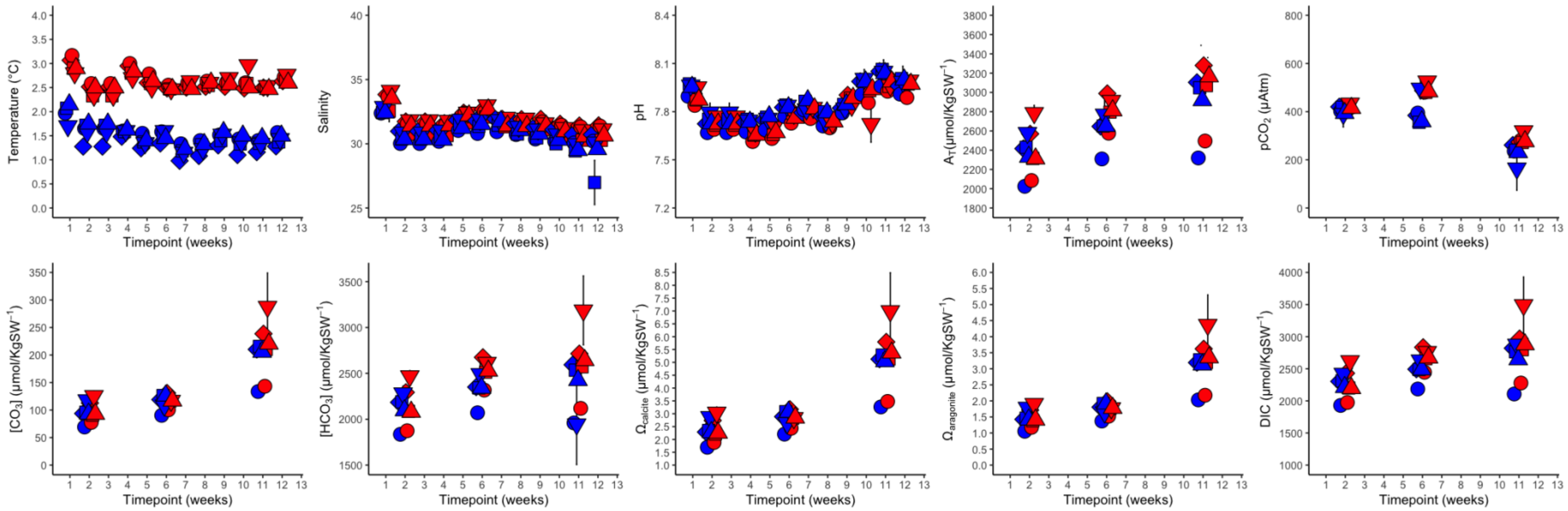

Figure S6: Classification of the experimental conditions for (a) *Astarte crenata* (b) *Ctenodiscus crispatus* (c) *Cistenides hyperborea* (d) *Aequiyoldia eightsi* and (e) *Laternula elliptica* over time (shape) under ambient (blue) and future (red) climate conditions. (a,c,d,e) shows a clear separation between treatment groups, with separation between treatments visible in week 11 for (b). Non-metric two-dimensional (nMDS) representations of euclidean similarity matrices based on 10 water and carbonate chemistry parameters (temperature, salinity, pH, total alkalinity, HCO_3, CO_3, pCO_2, calcite, aragonite and DIC) are presented. Dimensionality representation stress values (k=2) are (a) 0.034, (b) 0.021, (c) 0.015, (d) 0.006 and (e) 0.022. Permutational multivariate analyses of variance (PERMANOVA) outputs are provided.

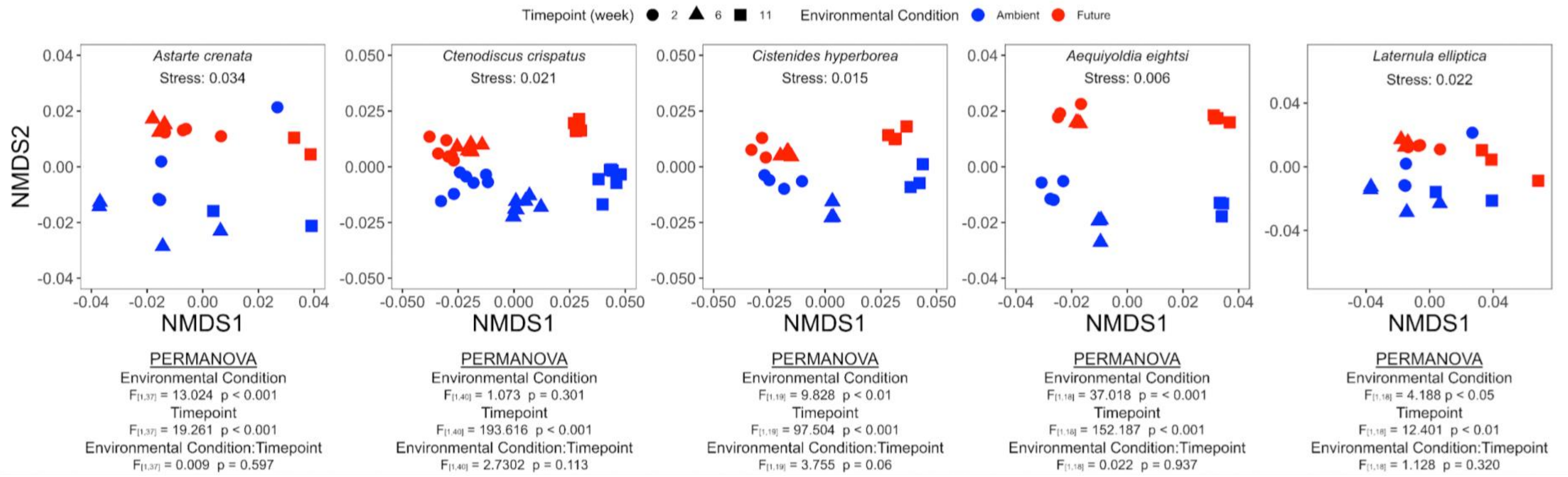

Table S5 (next three pages): Morphological and biomass measurements of (a) *Ctenodiscus crispatus* (b) *Cistenides hyperborea* (c) *Aequiyoldia eightsi* used for behaviour measurements.

Table S5(a)

Climate	Station	Arm length (mm)	Pit length (mm)	Biomass (g)
Ambient	B13	19.50	11.80	2.36
Ambient	B13	21.30	11.50	3.29
Ambient	B13	16.90	10.50	2.46
Ambient	B16	18.90	11.00	2.76
Ambient	B16	21.30	11.60	4.87
Ambient	B16	10.90	6.00	0.54
Ambient	B16	15.30	9.00	1.02
Ambient	B16	12.20	7.20	0.91
Future	B13	13.30	9.50	1.40
Future	B13	20.60	9.00	3.89
Future	B13	13.10	8.50	0.92
Future	B13	14.50	9.30	1.58
Future	B16	18.90	10.40	2.64
Future	B16	11.50	9.10	2.09
Future	B16	18.50	9.20	2.22
Future	B16	12.60	10.50	2.21
Future	B16	14.40	9.50	1.29
Future	B16	12.30	7.60	0.82

Table S5(b)

Climate	Station	Cone length (mm)	Anterior aperture (mm)	Biomass (g)
Ambient	B13	61.60	8.00	2.21
Ambient	B13	57.20	7.90	1.77
Ambient	B13	57.40	8.00	1.92
Ambient	B13	66.50	7.90	1.98
Ambient	B13	42.10	6.60	1.15
Future	B13	55.70	7.00	1.48
Future	B13	54.70	7.70	2.19
Future	B13	62.10	6.80	2.05
Future	B13	42.80	6.00	0.82
Future	B13	62.50	7.50	1.71
Future	B13	49.80	6.70	1.17

Table S5(c)

Climate	Shell Length (mm)	Shell Height (mm)	Shell Width (mm)	Biomass (g)
Ambient	22.40	19.70	6.20	1.52
Ambient	16.20	10.10	5.00	0.66
Ambient	18.80	12.40	5.90	0.99
Future	19.80	13.20	6.60	1.00
Future	24.00	14.70	6.60	1.77
Future	21.00	13.30	6.60	1.23

Figure S7: Selected time-lapse (3 frame s^{-1}, SkyStudioPro) images capturing movements of (a) *Aequiyoldia eightsi* (left viewing tray) and *Ctenodiscus crispatus* (right viewing tray) under future climate treatment (file: timelapse1.mp4), (b) *Ctenodiscus crispatus* (left viewing tray) and *Cistenides hyperborea* (right viewing tray) under ambient climate treatment (file: timelapse2.mp4). Frames are timestamped.

Figure S7(a)

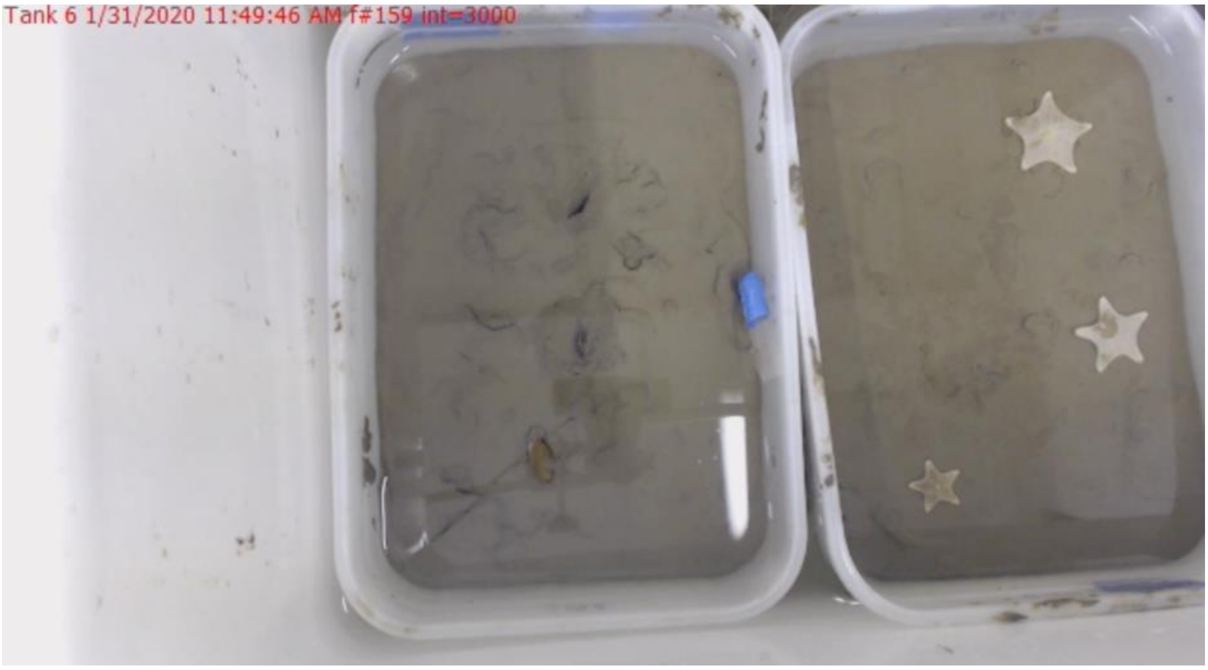

Figure S7(b)

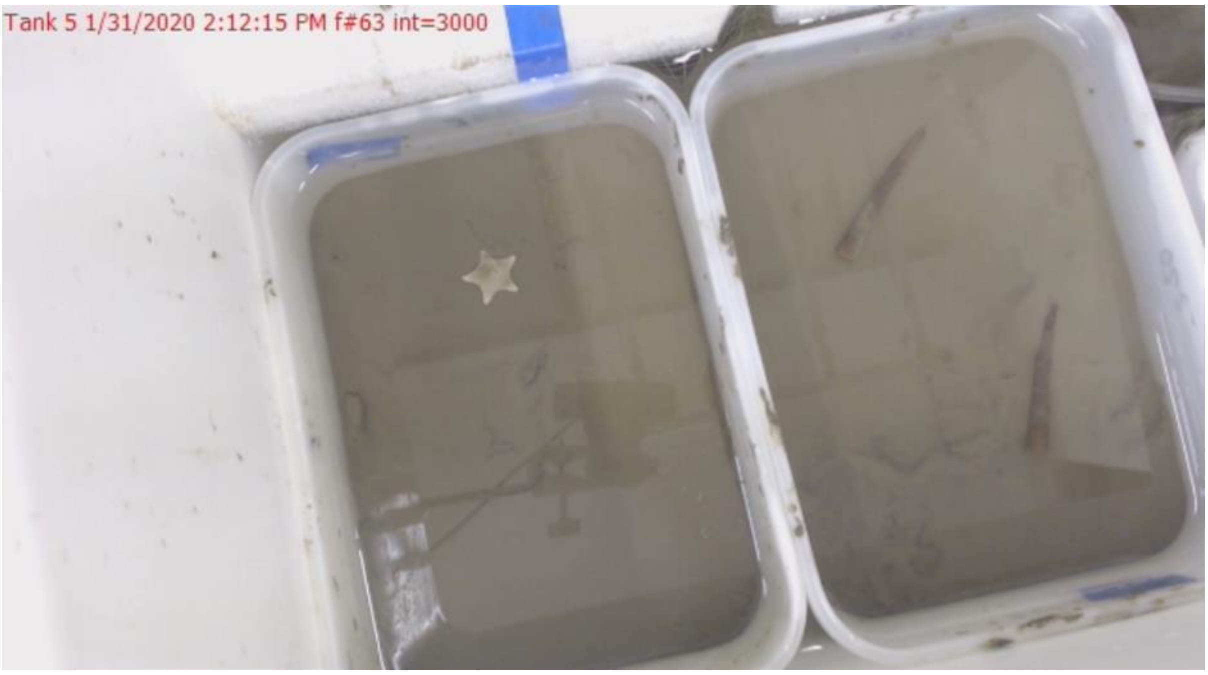

Figure S8 (next four pages): Replicate (n = 3) stitched f-SPI images for aquaria containing (a) *Astarte crenata* from (a) station B13 under ambient conditions (b) station B13 under future climate conditions (c) station B16 under ambient conditions and (d) station B16 under future climate conditions. The images (four aquarium sides, each 19cm, stitched together) are presented. The green coloration is the luminophore tracers after 10 days of incubation.

Figure S8(a)

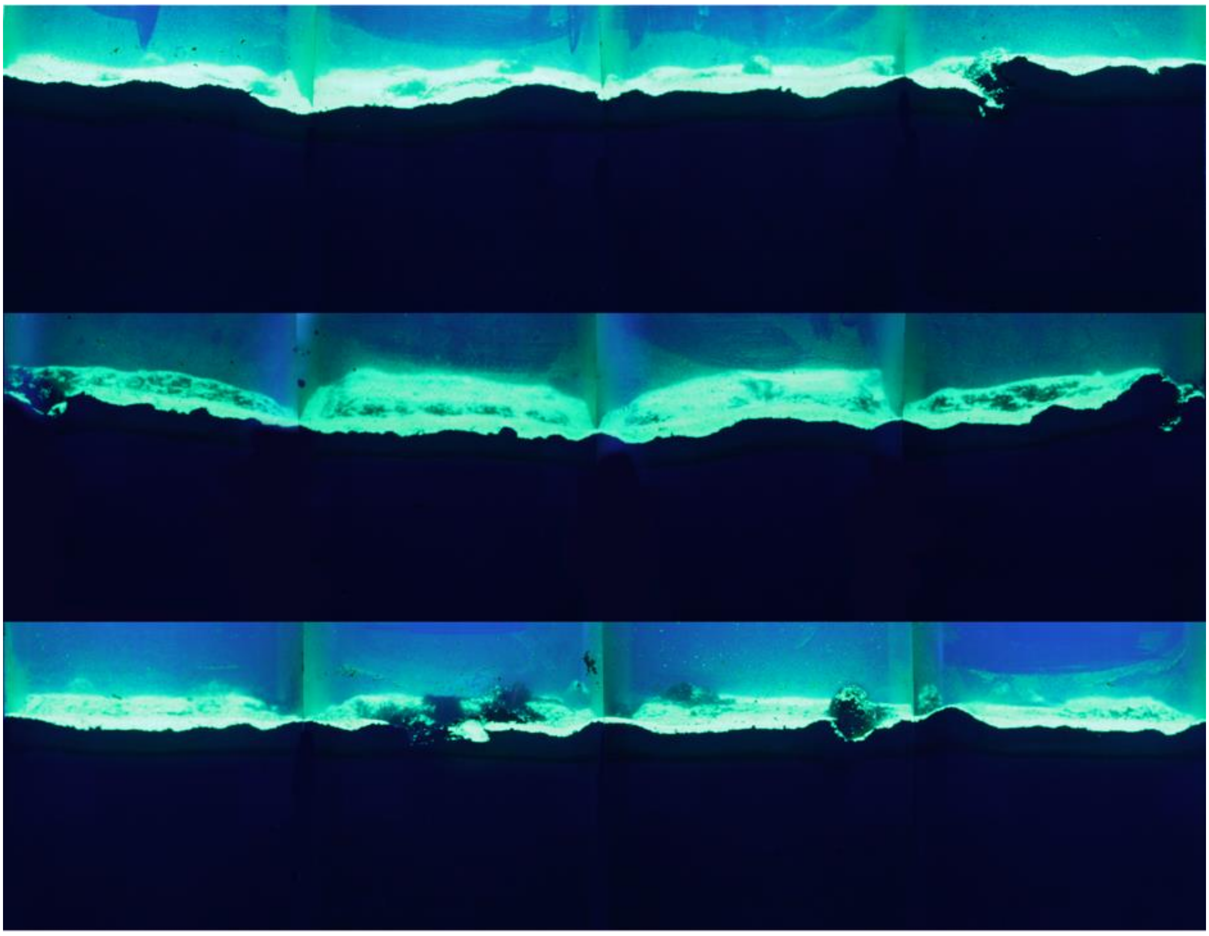

Figure S8(b)

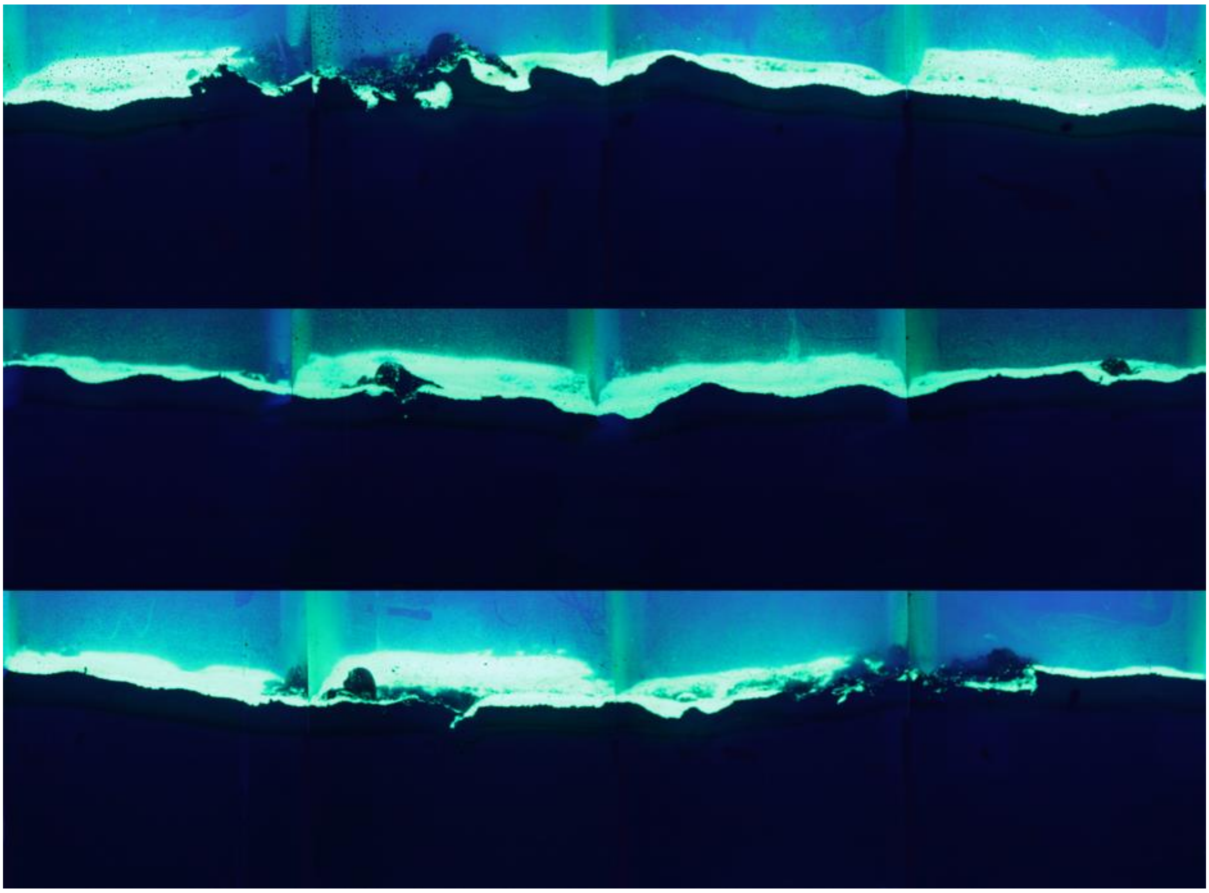

Figure S8(c)

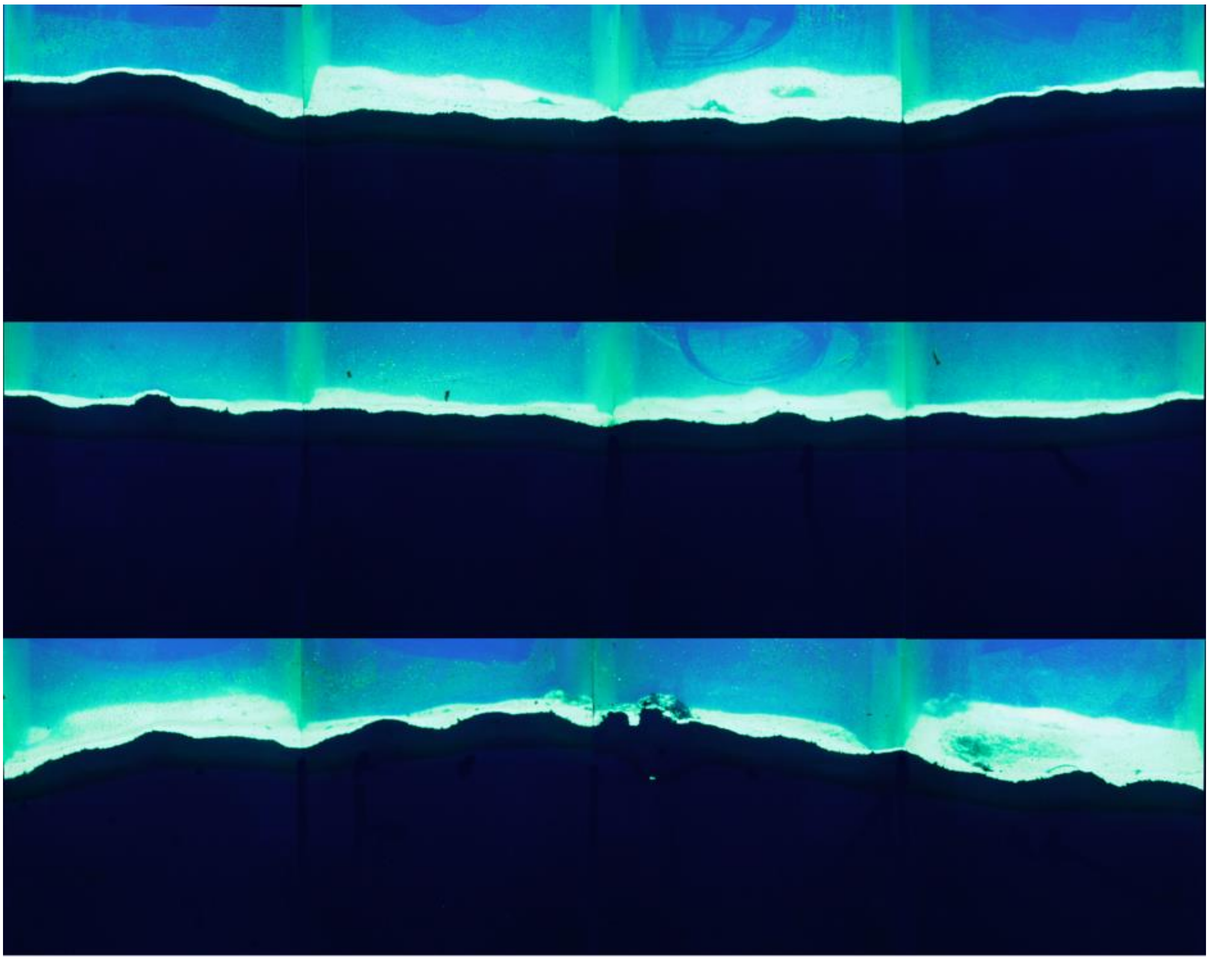

Figure S8(d)

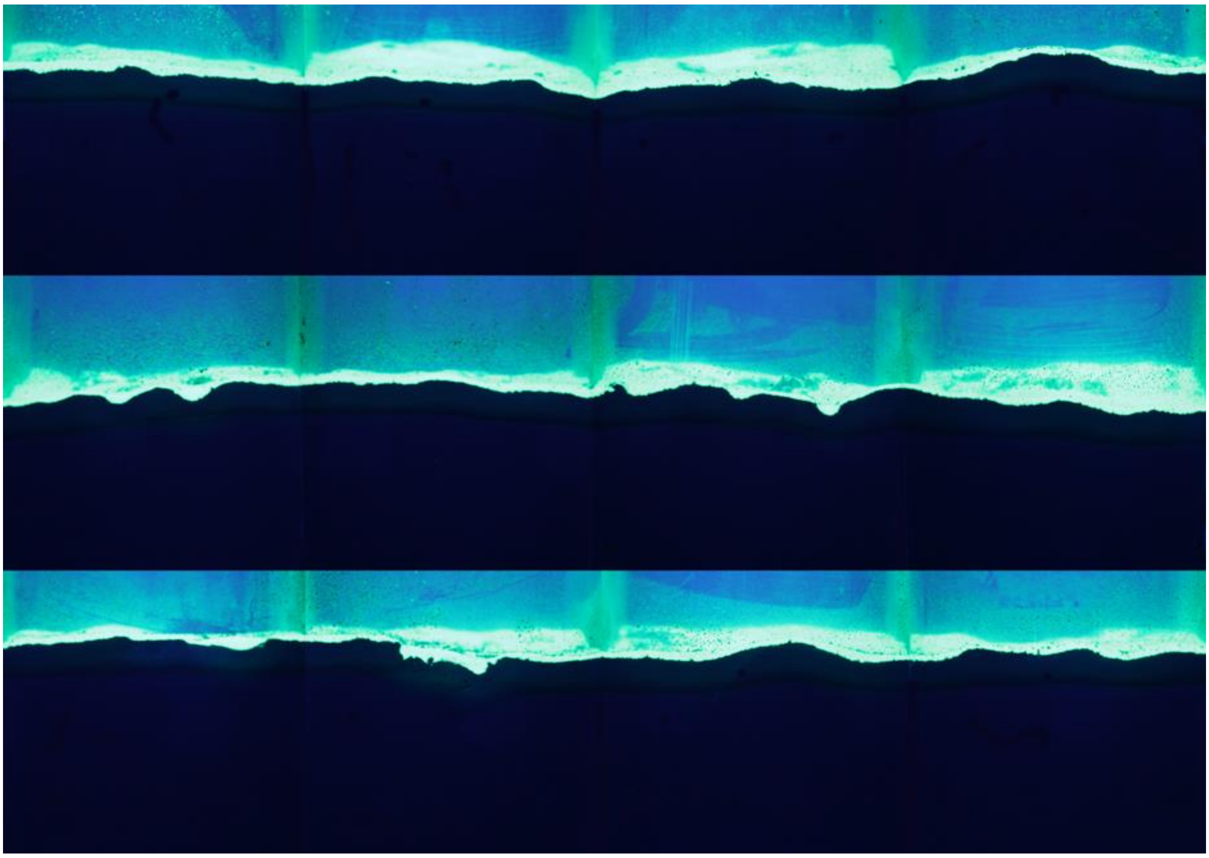

Figure S9 (next four pages): Replicate (n = 3) stitched f-SPI images for aquaria containing (a) *Ctenodiscus crispatus* from (a) station B13 under ambient conditions (b) station B13 under future climate conditions (c) station B16 under ambient conditions and (d) station B16 under future climate conditions. The images (four aquarium sides, each 19cm, stitched together) are presented. The green coloration is the luminophore tracers after 10 days of incubation.

Figure S9(a)

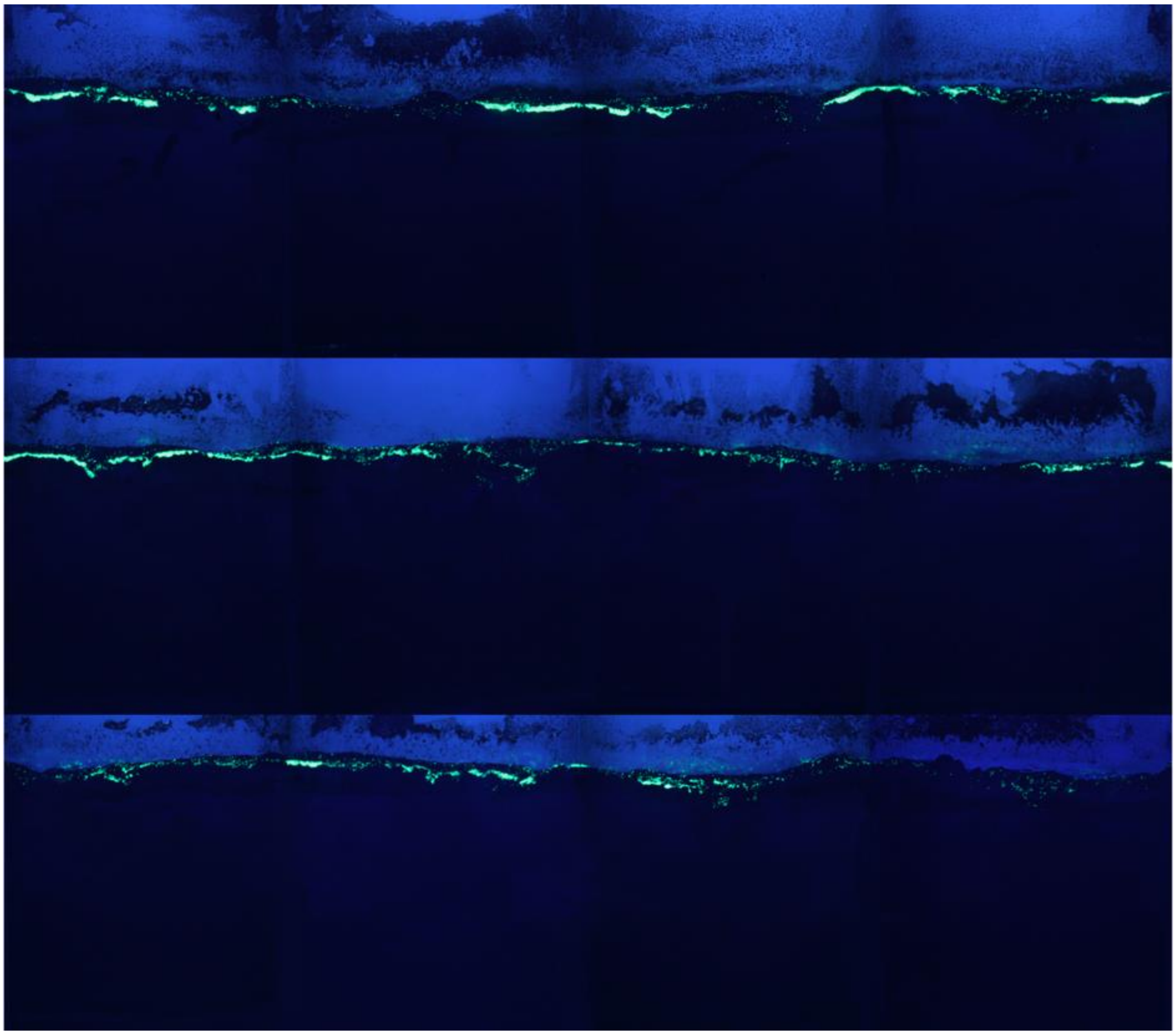

Figure S9(b)

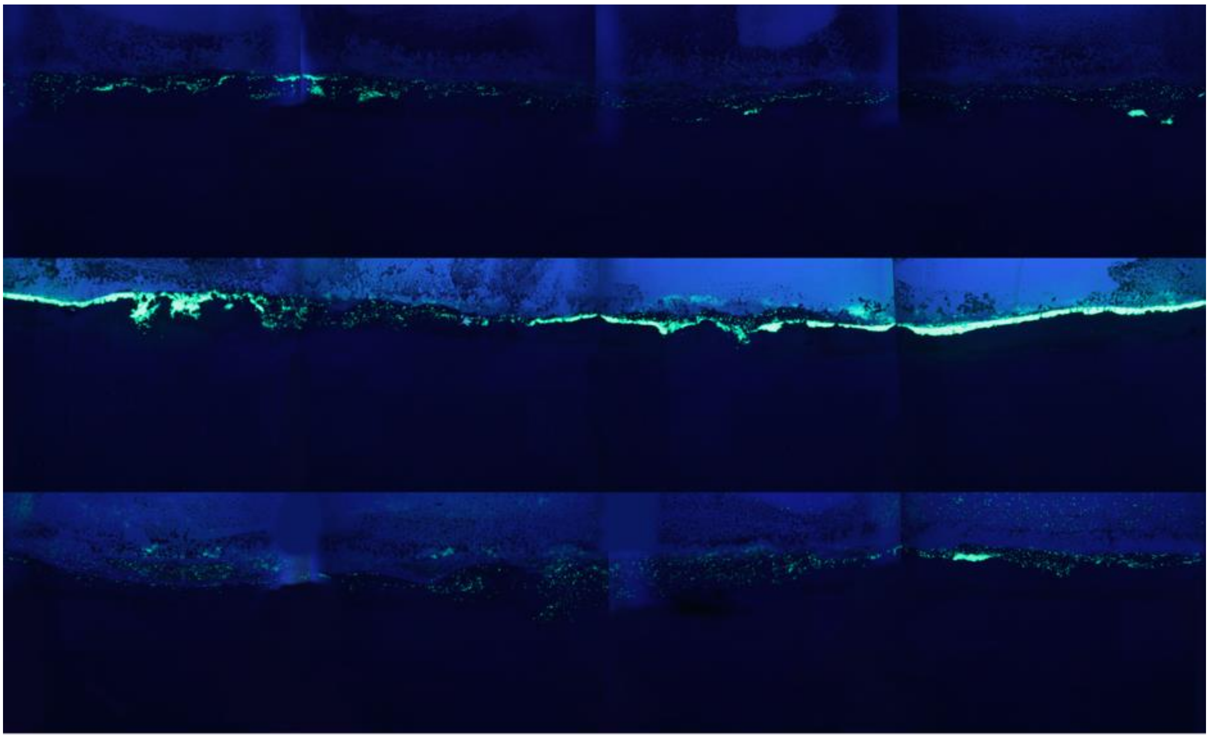

Figure S9(c)

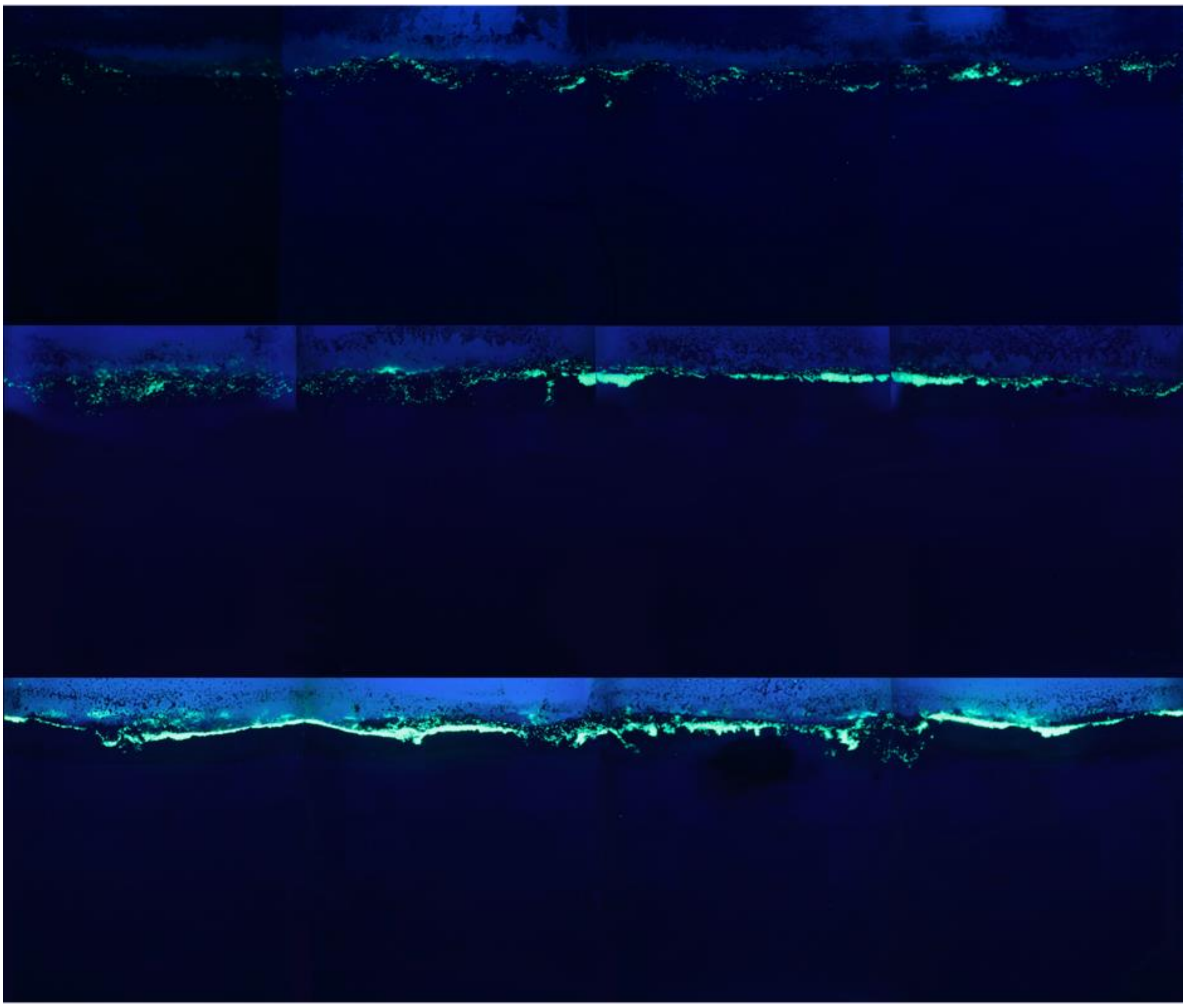

Figure S9(d)

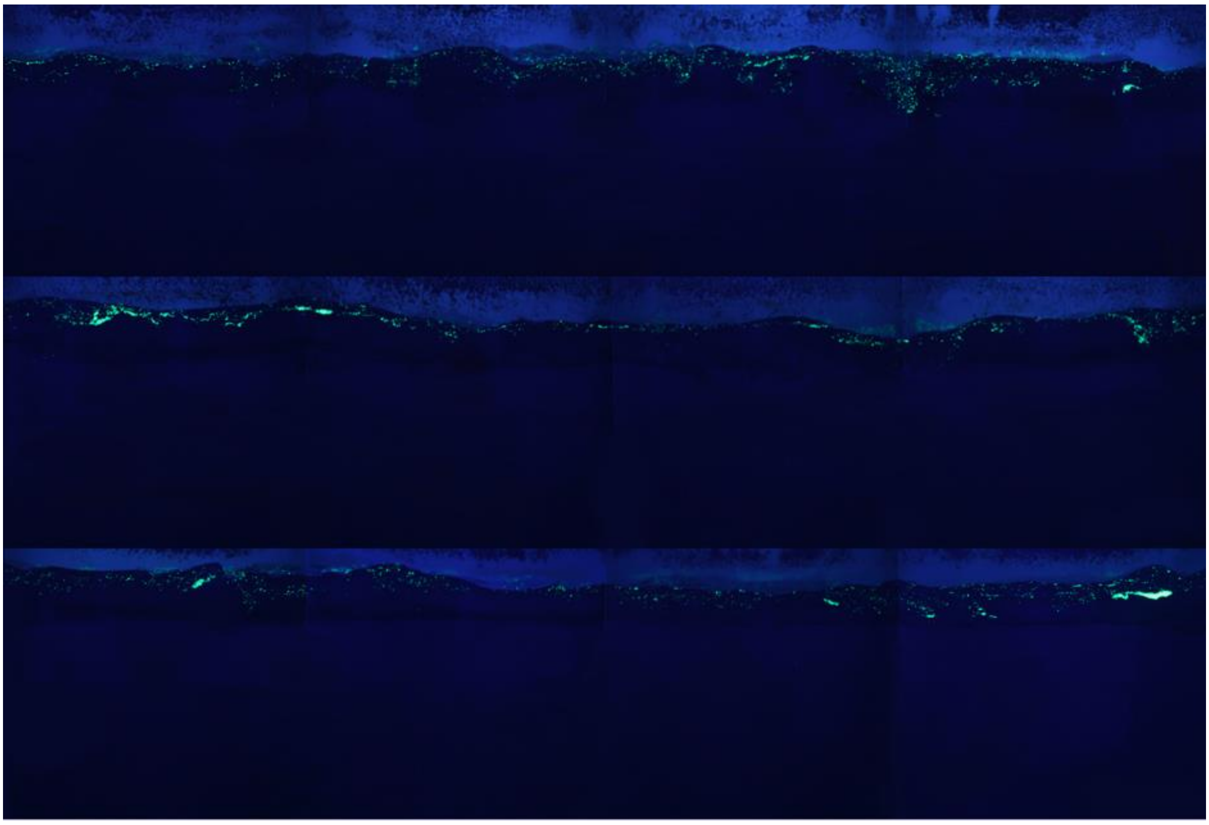

Figure S10 (next two pages): Replicate (n = 3) stitched f-SPI images for aquaria containing *Cistenides hyperborea* from (a) station B13 under ambient conditions (b) station B13 under future climate conditions. The images (four aquarium sides, each 19cm, stitched together) are presented. The green coloration is the luminophore tracers after 10 days of incubation.

Figure S10(a)

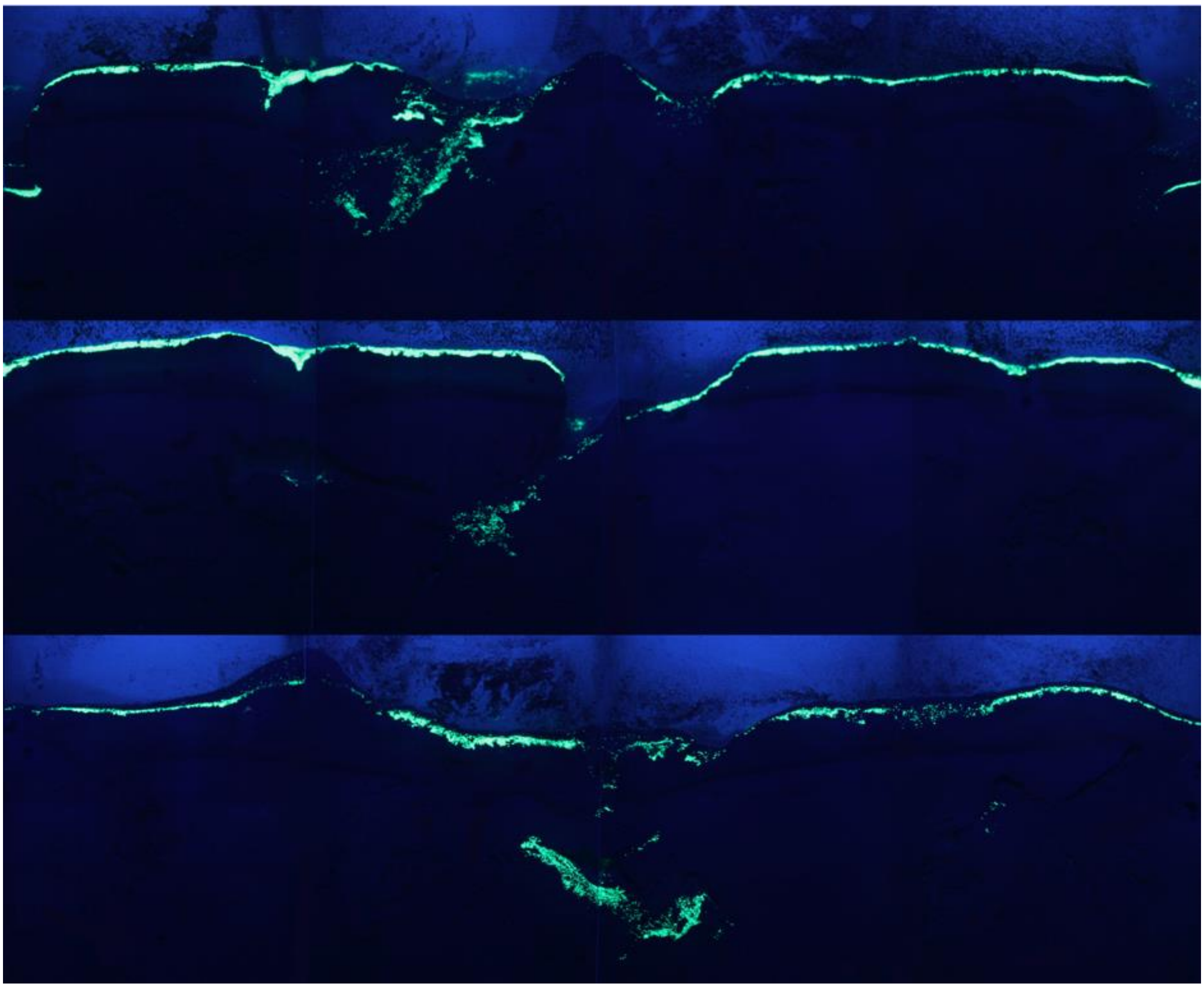

Figure S10(b)

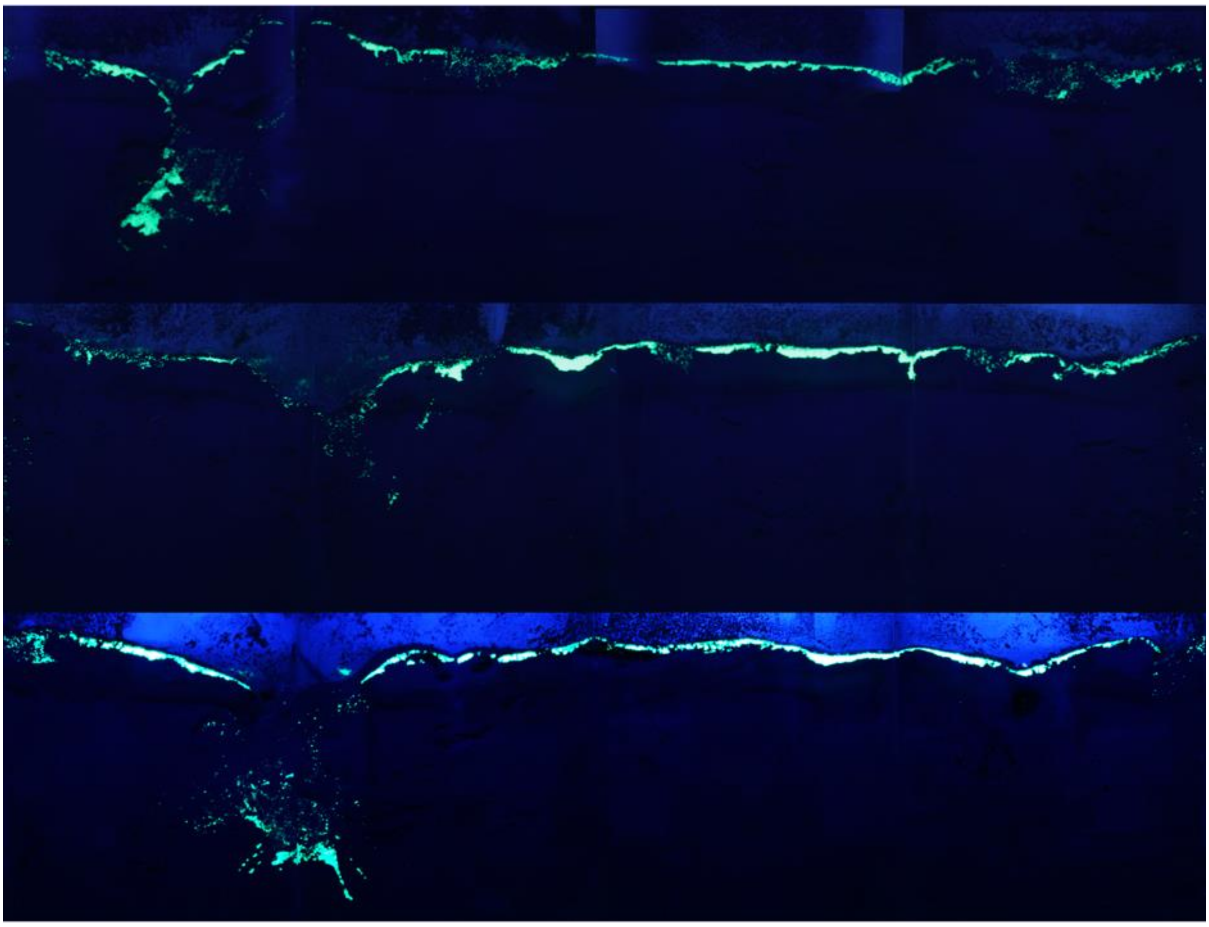

Figure S11 (next two pages): Replicate (n = 3) stitched f-SPI images for aquaria containing *Aequiyoldia eightsi* from (a) adjacent cove to Rothera research station under ambient conditions (b) adjacent cove to Rothera research station under future climate conditions. The images (four aquarium sides, each 19cm, stitched together) are presented. The green coloration is the luminophore tracers after 10 days of incubation.

Figure S11(a)

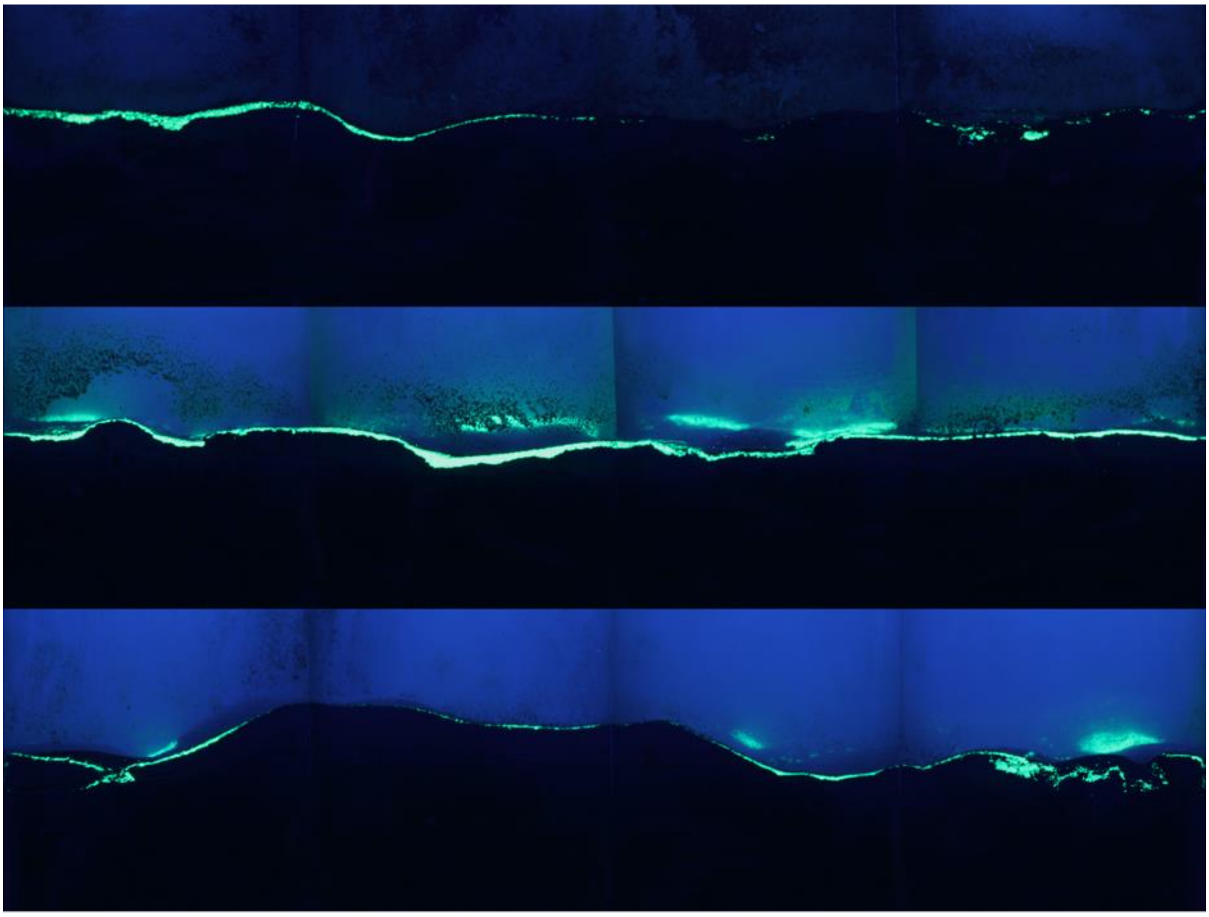

Figure S11(b)

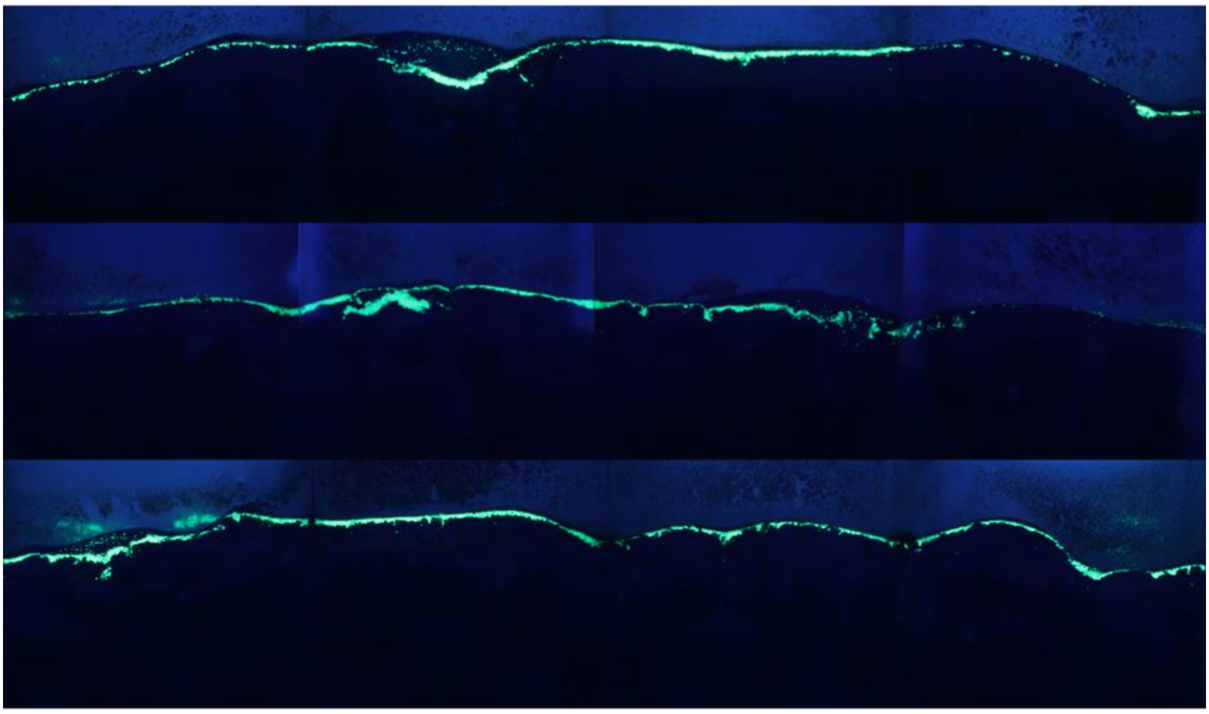

Figure S12 (next two pages): Replicate (n = 3) stitched f-SPI images for aquaria containing *Laternula elliptica* from (a) adjacent cove to Rothera research station under ambient conditions (b) adjacent cove to Rothera research station under future climate conditions. The images (four aquarium sides, each 19cm,stitched together) are presented. The green coloration is the luminophore tracers after 10 days of incubation.

Figure S12(a)

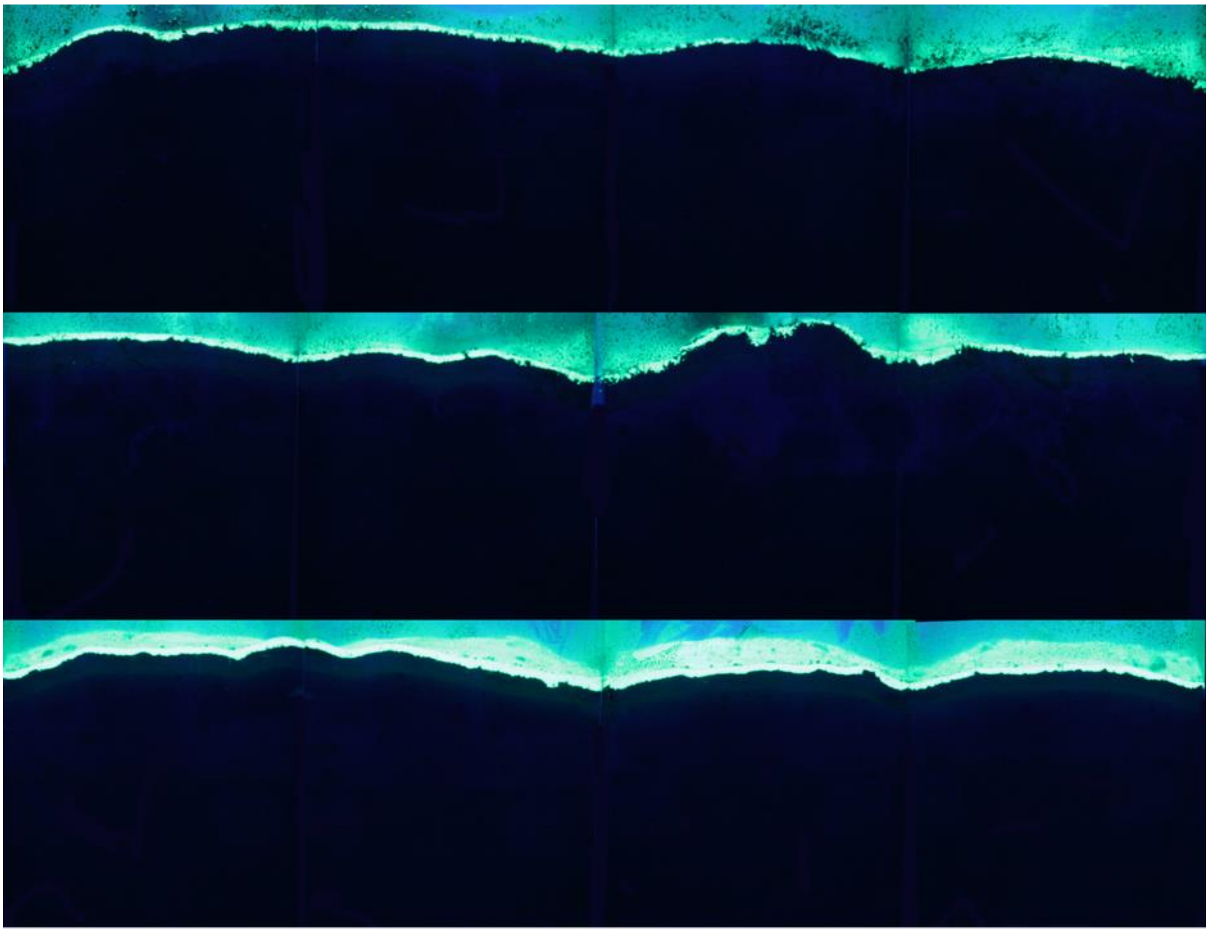

Figure S12(b)

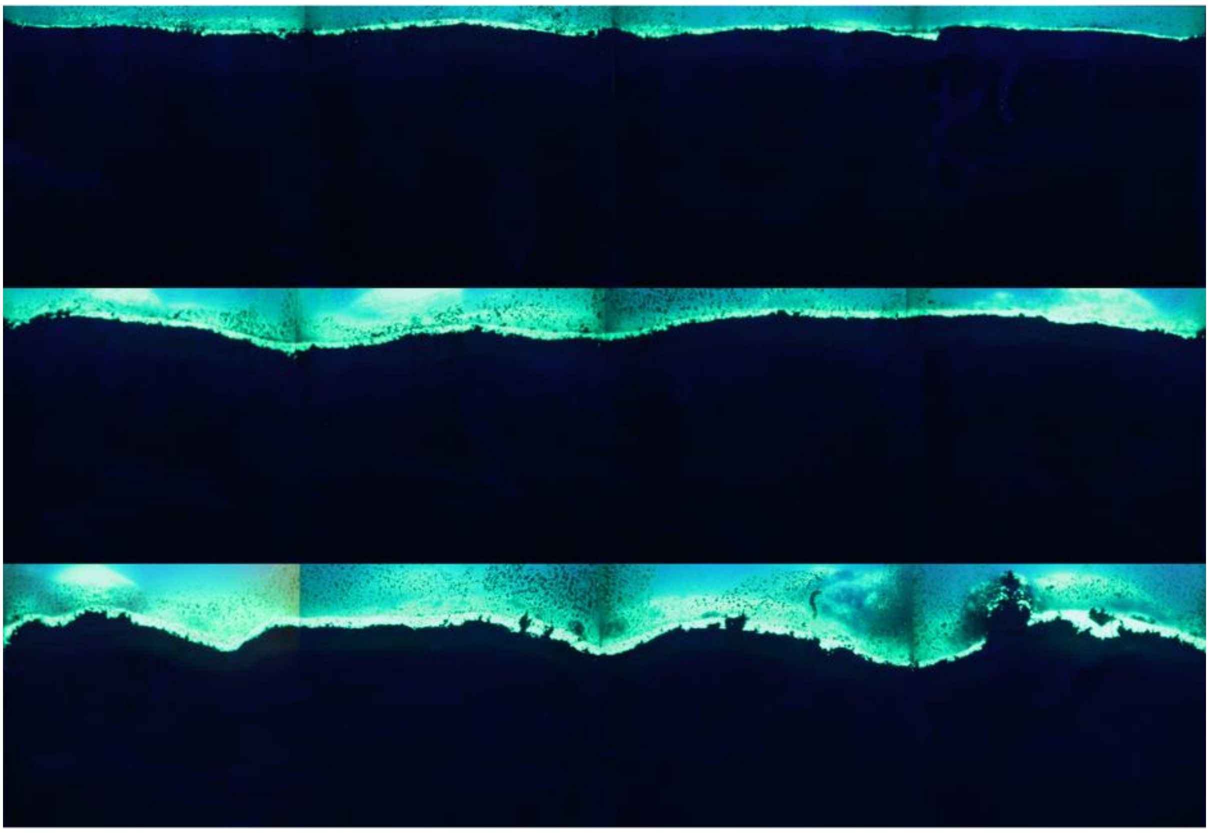

Figure S13 (next two pages): Sediment particle reworking profiles (n=3) derived from f-SPI images for (a) *Astarte crenata* from station B13 (b) *Astarte crenata* from B16 (c) *Ctenodiscus crispatus* from station B13 (d) *Ctenodiscus crispatus* from station B16 (e) *Cistenides hyperborea* from station B13 (f) *Aequiyoldia eightsi* and (g) *Laternula elliptica*. Line colour indicates environmental condition (ambient, black; future, red). Inserts show detail of the upper portion of the main figure.

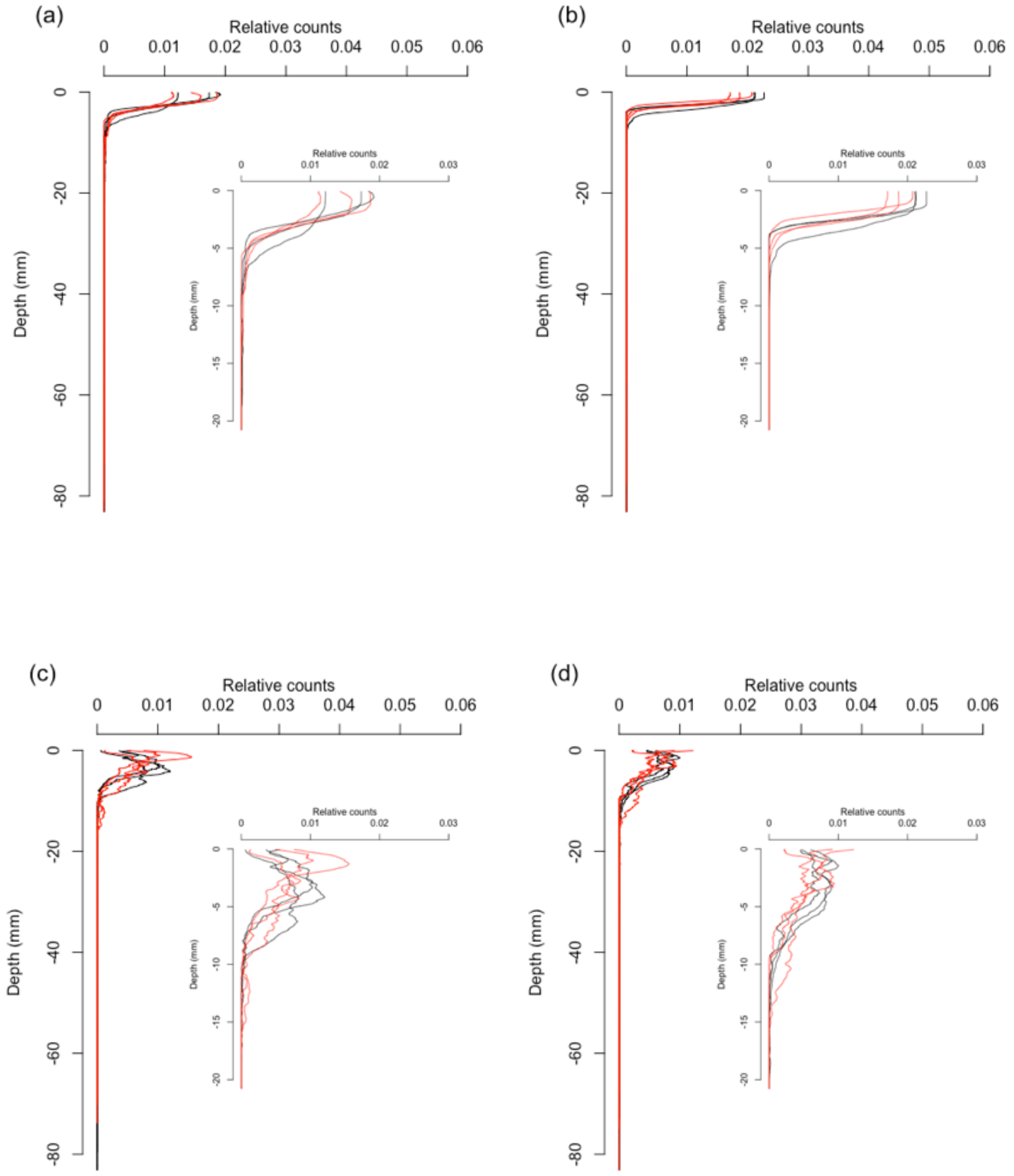

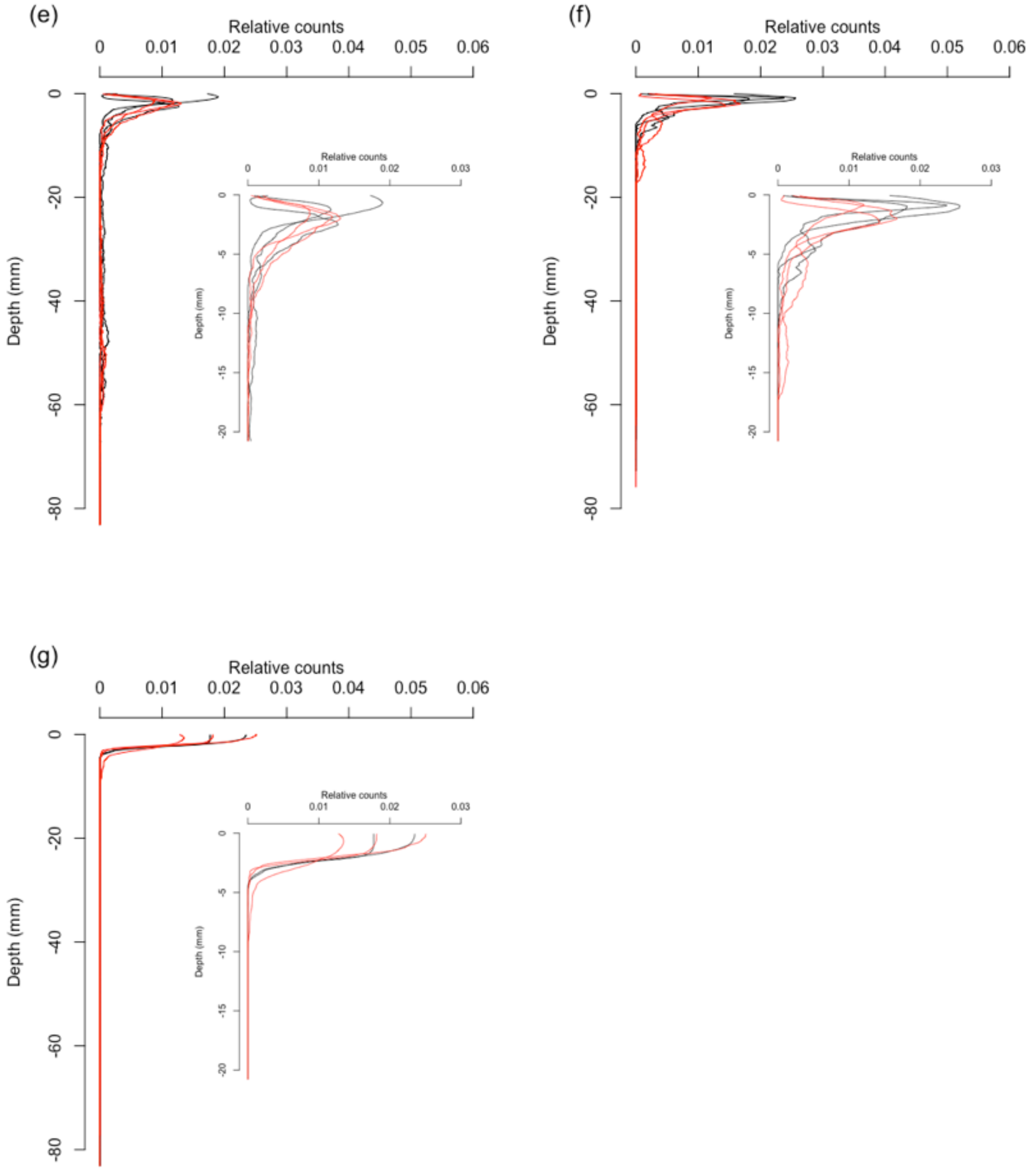
(e)
Relative counts
0 0.01 0.02 0.03 0.04 0.05 0.06
Depth (mm)
0 -20 -40 -60 -80
Relative counts
0 0.01 0.02 0.03
Depth (mm)
0 -5 -10 -15 -20
(f)
Relative counts
0 0.01 0.02 0.03 0.04 0.05 0.06
Depth (mm)
0 -20 -40 -60 -80
Relative counts
0 0.01 0.02 0.03
Depth (mm)
0 -5 -10 -15 -20
(g)
Relative counts
0 0.01 0.02 0.03 0.04 0.05 0.06
Depth (mm)
0 -20 -40 -60 -80
Relative counts
0 0.01 0.02 0.03
Depth (mm)
0 -5 -10 -15 -20

Figure S14: Monthly nutrient ([NH4-N];[NO2-N];[NO3-N];[PO4-P]) concentrations in aquaria maintained under ambient (1 °C, 400 ppm [CO_2]; open symbols) and future (2.5 °C, 550 ppm [CO_2]; closed symbols) environmental conditions containing *Astarte crenata* (circles) and *Ctenodiscus crispatus* (squares) from station B13 (red symbols) and B16 (blue symbols); *Cistenides hyperborea* (triangles); *Aequiyoldia eightsi* (diamonds) and *Laternula eightsi* (inverted triangles).

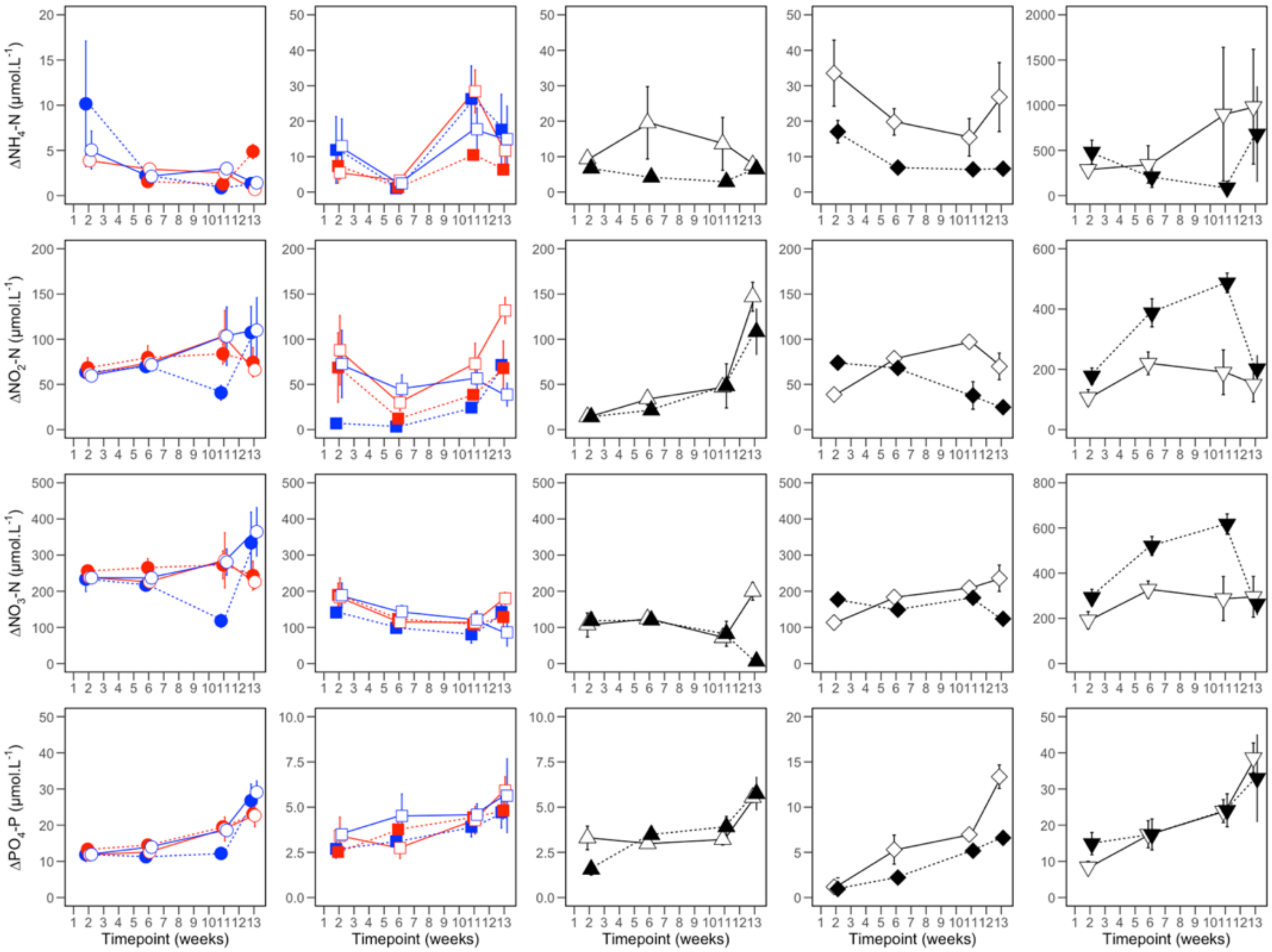

Statistical model summary

Summary of the statistical models analysing each species group (Arctic: *A. crenata* & *C. crispat*us; *C. hyperborea*; Antarctic: *A. eightsi* & *L. elliptica*) separately (Model S1 to S29). For each model we list the initial linear regression model and the minimal adequate model. When homogeneity of variance was violated we used a linear regression with generalised least squares (GLS) estimation. We present a summary of the coefficient tables for single terms. The coefficients indicate the relative performance of each factor level in relation to the re-levelled baseline (as indicated). Coefficients ± SE, t-values and respective significance values are presented.

Abbreviations

(i) Explanatory variables

Climate, environmental condition
Station, cruise station
SPID, species Identity

(ii) Response variables

Response Time, time to initiate movement (s)
Burial Time, time to complete burial (s)
SBR, surface boundary roughness (mm)
$^{f\text{-}SPI}L_{median,}$, median mixed depth of particle reworking (mm)
$^{f\text{-}SPI}L_{max,}$, maximum mixed depth of particle reworking (mm)
$\Delta[Br^-]$, burrow ventilation (mg L^{-1})
$\Delta[NH_4\text{-}N]$, NH_4-N (lnRR)
$\Delta[NO_2\text{-}N]$, NO_2-N (lnRR)
$\Delta[NO_3\text{-}N]$, NO_3-N (lnRR)
$\Delta[PO_4\text{-}P]$, PO_4-P (lnRR)

Data: All data used in the analyses are provided as Table S6.

Arctic species

Model S1 Time to initiate movement (Response Time, s) - *Ctenodiscus crispatus*

Initial linear regression model:

```
lm(Response Time ~ Climate + Station + Climate:Station)
```

Minimal adequate model:

```
gls(Response Time ~ 1, weights = varIdent(form = ~ 1|Station),
method = "REML")
```

Model S2 Time to complete burial (Burial Time, s) - *Ctenodiscus crispatus*

Initial linear regression model:

```
lm(Burial Time ~ Climate + Station + Climate:Station)
```

Minimal adequate model:

```
lm(Burial Time ~ Climate)
```

Intercept ± SE (when baseline is for Ambient for Climate): 995.0 ± 160.6, t = 6.1978, **p < 0.0001**

Coefficient table for Climate

	Ambient	Future
Ambient	/	-497.1 ± 203.1<br>-2.448<br>**0.0282**
Future	-497.1 ± 203.1<br>-2.448<br>**0.0282**	/

Model S3 Surface boundary roughness (SBR, mm) – *Astarte crenata* Ctenodiscus crispatus*

Initial linear regression model:

```
lm(SBR ~ Climate + Station + SPID + Climate:Station +
Climate:SPID + Station:SPID + Climate:Station:SPID)
```

Minimal adequate model:

```
gls(SBR ~ Station + SPID, weights = varIdent(form = ~1|SPID),
method = "REML")
```

Intercept ± SE (when baseline is for B13 for Station and *A. crenata* for SPID): 17.452 ± 1.701. t = 10.259, **p = 0**

Coefficient table for Station

	B13	B16
B13	/	-2.096 ± 1.045<br>-2.007<br>0.058
B16	-2.096 ± 1.045<br>-2.007<br>0.058	/

Coefficient table for SPID

	Astarte crenata	*Ctenodiscus crispatus*
Astarte crenata	/	-6.261 ± 1.709<br>-3.663<br>**0.002**
Ctenodiscus crispatus	-6.261 ± 1.709<br>-3.663<br>**0.002**	/

Model S4 Median mixing depth ($^{f\text{-}SPI}L_{median}$, mm) – *Astarte crenata* Ctenodiscus crispatus*

Initial linear regression model:

`lm(`$^{f\text{-}SPI}L_{median}$` ~ Climate + Station + SPID + Climate:Station + Climate:SPID + Station:SPID + Climate:Station:SPID)`

Minimal adequate model:

`lm(`$^{f\text{-}SPI}L_{median}$` ~ Climate + Station + SPID + Climate:SPID + Station:SPID)`

Model S5 Maximum mixing depth ($^{f\text{-}SPI}L_{max}$, mm) – *Astarte crenata* Ctenodiscus crispatus*

Initial linear regression model:

`lm(`$^{f\text{-}SPI}L_{max}$` ~ Climate + Station + SPID + Climate:Station + Climate:SPID + Station:SPID + Climate:Station:SPID)`

Minimal adequate model:

`lm(`$^{f\text{-}SPI}L_{max}$` ~ Station + SPID + Station:SPID)`

Intercept ± SE: 5.714 ± 1.875, t = 3.052, **p < 0.01**

Model S6 Burrow ventilation (Δ[Br$^-$], mg L^{-1}) – *Astarte crenata* Ctenodiscus crispatus*

Initial linear regression model:

```
lm(Δ[Br⁻] ~ Climate + Station + SPID + Climate:Station +
Climate:SPID + Station:SPID + Climate:Station:SPID)
```

Minimal adequate model:

```
lm(Δ[Br⁻] ~ Climate + Station + SPID + Climate:Station +
Climate:SPID + Station:SPID + Climate:Station:SPID)
```

Model S7 NH_4-N flux (Δ[NH_4-N], lnRR) – *Astarte crenata* Ctenodiscus crispatus*

Initial linear regression model:

```
lm(Δ[NH4-N] ~ Climate + Station + SPID + Climate:Station +
Climate:SPID + Station:SPID + Climate:Station:SPID)
```

Minimal adequate model:

```
lm(Δ[NH4-N] ~ SPID)
```

Intercept ± SE (when baseline is for *Astarte crenata* for SPID): -0.636 ± 0.147, t = -4.336, **p < 0.001**

Coefficient table for Climate

	Astarte crenata	*Ctenodiscus crispatus*
Astarte crenata	/	0.802 ± 0.207<br>3.867<br>**<0.001**
Ctenodiscus crispatus	0.802 ± 0.207<br>3.867<br>**<0.001**	/

Model S8 NO_2-N flux (Δ[NO_2-N], lnRR) – *Astarte crenata* Ctenodiscus crispatus*

Initial linear regression model:

```
lm(Δ[NO2-N] ~ Climate + Station + SPID + Climate:Station +
Climate:SPID + Station:SPID + Climate:Station:SPID)
```

Minimal adequate model:

```
gls(Δ[NO2-N] ~ Climate + Station + SPID + Climate:Station +
Climate:SPID + Station:SPID + Climate:Station:SPID, weights =
= varIdent(form = ~1|SPID), method = "REML")
```

Model S9 NO_3-N flux (Δ[NO_3-N], lnRR) – *Astarte crenata* Ctenodiscus crispatus*

Initial linear regression model:

```
lm(Δ[NO3-N] ~ Climate + Station + SPID + Climate:Station +
Climate:SPID + Station:SPID + Climate:Station:SPID)
```

Minimal adequate model:

```
lm(Δ[NO3-N] ~ Climate + Station + SPID + Climate:Station +
Climate:SPID + Station:SPID + Climate:Station:SPID)
```

Model S10 PO_4-P flux (Δ[PO_4-P], lnRR) – *Astarte crenata* Ctenodiscus crispatus*

Initial linear regression model:

```
lm(Δ[PO4-P] ~ Climate + Station + SPID + Climate:Station +
Climate:SPID + Station:SPID + Climate:Station:SPID)
```

Minimal adequate model:

```
lm(Δ[PO4-P] ~ 1)
```

Model S11 Time to initiate movement (Response Time, s) - *Cistenides hyperborea*

Initial linear regression model:

```
lm(Response Time ~ Climate)
```

Minimal adequate model:

```
lm(Response Time ~ 1)
```

Model S12 Surface boundary roughness (SBR, mm) – *Cistenides hyperborea*

Initial linear regression model:

lm(SBR ~ Climate)

Minimal adequate model:

gls(SBR ~ 1, weights = varIdent(form = ~1|Climate), method = "REML")

Model S13 Median mixing depth ($^{f\text{-}SPI}L_{median}$, mm) – *Cistenides hyperborea*

Initial linear regression model:

lm($^{f\text{-}SPI}L_{median}$ ~ Climate)

Minimal adequate model:

gls($^{f\text{-}SPI}L_{median}$ ~ 1, weights = varIdent(form = ~1|Climate), method = "REML")

Model S14 Maximum mixing depth ($^{f\text{-}SPI}L_{max}$, mm) – *Cistenides hyperborea*

Initial linear regression model:

lm($^{f\text{-}SPI}L_{max}$ ~ Climate)

Minimal adequate model:

gls($^{f\text{-}SPI}L_{max}$ ~ 1, weights = varIdent(form = ~1|Climate), method = "REML")

Model S15 Burrow ventilation ($\Delta[Br^-]$, mg L^{-1}) – *Cistenides hyperborea*

Initial linear regression model:

lm($\Delta[Br^-]$ ~ Climate)

Minimal adequate model:

gls($\Delta[Br^-]$ ~ Climate, weights = varIdent(form = ~1|Climate), method = "REML")

Intercept ± SE (when baseline is for Ambient for Climate):

71.611 ± 178.821, $t = 0.400$, $p = 0.709$

Coefficient table for Climate

	Ambient	Future

Ambient	/	-634.850 ± 198.567<br>-3.197<br>**0.033**
Future	634.850 ± 198.567<br>-3.197<br>**0.033**	/

Model S16 NH_4-N flux (Δ[NH_4-N], lnRR) – *Cistenides hyperborea*

Initial linear regression model:

`lm(Δ[NH4-N] ~ Climate)`

Minimal adequate model:

`lm(Δ[NH4-N] ~ 1)`

Model S17 NO_2-N flux (Δ[NO_2-N], lnRR) – *Cistenides hyperborea*

Initial linear regression model:

`lm(Δ[NO2-N] ~ Climate)`

Minimal adequate model:

`lm(Δ[NO2-N] ~ 1)`

Model S18 NO_3-N flux (Δ[NO_3-N], lnRR) – *Cistenides hyperborea*

Initial linear regression model:

`lm(Δ[NO3-N] ~ Climate)`

Minimal adequate model:

`lm(Δ[NO3-N] ~ Climate)`

Intercept ± SE (when baseline is for Ambient for Climate):

0.325 ± 0.143, t = 2.273, p = 0.085

Coefficient table for Climate

	Ambient	Future
Ambient	/	-1.574 ± 0.202<br>-7.799<br>**0.002**
Future	-1.574 ± 0.202<br>-7.799	/

	**0.002**	

Model S19 PO_4-P flux ($\Delta[PO_4$-P], lnRR) – *Cistenides hyperborea*

Initial linear regression model:

```
lm(Δ[PO4-P] ~ Climate)
```

Minimal adequate model:

```
gls(Δ[PO4-P] ~ 1, weights = varIdent(form = ~1|Climate), method
= "REML")
```

Antarctic species

Model S20 Time to initiate movement (Response Time, s) - *Aequiyoldia eightsi*

Initial linear regression model:

```
lm(Response Time ~ Climate)
```

Minimal adequate model:

```
gls(Response Time ~ 1, weights = varIdent(form = ~1|Climate),
method = "REML")
```

Model S21 Time to complete burial (Burial Time, s) - *Aequiyoldia eightsi*

Initial linear regression model:

```
lm(Response Time ~ Climate)
```

Minimal adequate model:

```
gls(Response Time ~ 1, weights = varIdent(form = ~1|Climate),
method = "REML")
```

Model S22 Surface boundary roughness (SBR, mm) – *Laternula elliptica*Aequiyoldia eightsi*

Initial linear regression model:

```
lm(SBR ~ Climate + SPID + Climate:SPID)
```

Minimal adequate model:

lm(SBR ~ Climate + SPID + Climate:SPID)

Model S23 Median mixing depth ($^{f\text{-}SPI}L_{median}$, mm) – *Laternula elliptica*Aequiyoldia eightsi*

Initial linear regression model:

lm($^{f\text{-}SPI}L_{median}$ ~ Climate + SPID + Climate:SPID)

Minimal adequate model:

lm($^{f\text{-}SPI}L_{median}$ ~ Climate + SPID + Climate:SPID)

Model S24 Maximum mixing depth ($^{f\text{-}SPI}L_{max}$, mm) – *Laternula elliptica*Aequiyoldia eightsi*

Initial linear regression model:

lm($^{f\text{-}SPI}L_{max}$ ~ Climate + SPID + Climate:SPID)

Minimal adequate model:

lm($^{f\text{-}SPI}L_{max}$ ~ Climate + SPID + Climate:SPID)

Model S25 Burrow ventilation (Δ[Br^-], mg L^{-1}) – *Laternula elliptica*Aequiyoldia eightsi*

Initial linear regression model:

lm(Δ[Br^-] ~ Climate + SPID + Climate:SPID)

Minimal adequate model:

gls(Δ[Br^-] ~ 1, weights = varIdent(form = ~1|SPID), method = "REML")

Model S26 NH_4-N flux (Δ[NH_4-N], lnRR) – *Laternula elliptica*Aequiyoldia eightsi*

Initial linear regression model:

lm(Δ[NH_4-N] ~ Climate + SPID + Climate:SPID)

Minimal adequate model:

gls(Δ[NH_4-N] ~ 1, weights = varIdent(form = ~ 1|SPID*Climate, method = "REML")

Model S27 [NO_2-N] flux (Δ[NO_2-N], lnRR) – *Laternula elliptica*Aequiyoldia eightsi*

Initial linear regression model:

```
lm(Δ[NO2-N] ~ Climate + SPID + Climate:SPID)
```

Minimal adequate model:

```
gls(Δ[NO2-N] ~ 1, weights = varIdent(form = ~ 1|SPID*Climate,
method = "REML")
```

Model S28 NO_3-N flux (Δ[NO_3-N], lnRR) – *Laternula elliptica*Aequiyoldia eightsi*

Initial linear regression model:

```
lm(Δ[NO3-N] ~ Climate + SPID + Climate:SPID)
```

Minimal adequate model:

```
lm(Δ[NO3-N] ~ Climate, weights = varIdent(form = ~
1|SPID*Climate, method = "REML")
```

Intercept ± SE (when baseline is for Ambient for Climate and *Laternula elliptica* for SPID):

0.295 ± 0.077, t = 3.810, **p < 0.01**

Coefficient table for Climate

	Ambient	Future
Ambient	/	-0.451 ± 0.078<br>-5.801<br>**<0.001**
Future	-0.451 ± 0.078<br>-5.801<br>**<0.001**	/

Model S29 PO_4-P flux (Δ[PO_4-P], lnRR) – *Laternula elliptica*Aequiyoldia eightsi*

Initial linear regression model:

```
lm(Δ[PO4-P] ~ Climate + SPID + Climate:SPID)
```

Minimal adequate model:

```
gls(Δ[PO4-P] ~ Climate + SPID, weights = varIdent(form =
~1|SPID*Climate), method = "REML")
```

Intercept ± SE (when baseline is Ambient for Climate and *Laternula elliptica* for SPID):

0.676 ± 0.068, t = 9.934, **p < 0.0001**

Coefficient table for Climate

	Ambient	Future
Ambient	/	-0.369 ± 0.096<br>-3.861<br>**<0.01**
Future	-0.369 ± 0.096<br>-3.861<br>**<0.01**	/

Coefficient table for SPID

	Laternula elliptica	*Aequiyoldia eightsi*
Laternula elliptica	/	0.655 ± 0.313<br>2.089<br>0.066
Aequiyoldia eightsi	0.655 ± 0.313<br>2.089<br>0.066	/

Table S6 (the next three pages): Summary of (a) intraspecific behavioural activity and (b) ecosystem process and functioning used in our statistical analyses. Climate = environmental condition (ambient vs future), Station = location (B13 = station B13, B16 = station B16).
* indicates individuals that did not respond within our maximum observation period (3600s).

Table S6(a)

Climate	Station	Species identity	Replicate	Response Time (s)	Burial Time (s)
Ambient	B13	*Ctenodiscus crispatus*	1	215	683
Ambient	B13	*Ctenodiscus crispatus*	2	512	1722
Ambient	B13	*Ctenodiscus crispatus*	3	336	670
Ambient	B16	*Ctenodiscus crispatus*	1	122	1480
Ambient	B16	*Ctenodiscus crispatus*	2	253	*
Ambient	B16	*Ctenodiscus crispatus*	3	722	886
Ambient	B16	*Ctenodiscus crispatus*	4	1026	*
Ambient	B16	*Ctenodiscus crispatus*	5	385	529
Ambient	B13	*Cistenides hyperborea*	1	787	*
Ambient	B13	*Cistenides hyperborea*	2	1577	*
Ambient	B13	*Cistenides hyperborea*	3	731	*
Ambient	B13	*Cistenides hyperborea*	4	293	*
Ambient	Rothera	*Aequiyoldia eightsi*	1	529	175
Ambient	Rothera	*Aequiyoldia eightsi*	2	1322	184
Ambient	Rothera	*Aequiyoldia eightsi*	3	97	74
Future	B13	*Ctenodiscus crispatus*	1	296	575
Future	B13	*Ctenodiscus crispatus*	2	548	1331
Future	B13	*Ctenodiscus crispatus*	3	260	231
Future	B13	*Ctenodiscus crispatus*	4	429	222
Future	B16	*Ctenodiscus crispatus*	1	386	318
Future	B16	*Ctenodiscus crispatus*	2	458	682
Future	B16	*Ctenodiscus crispatus*	3	631	459
Future	B16	*Ctenodiscus crispatus*	4	978	486
Future	B16	*Ctenodiscus crispatus*	5	173	377
Future	B16	*Ctenodiscus crispatus*	6	281	298
Future	B13	*Cistenides hyperborea*	1	193	1034
Future	B13	*Cistenides hyperborea*	2	1263	*
Future	B13	*Cistenides hyperborea*	3	538	2376
Future	B13	*Cistenides hyperborea*	4	1309	*
Future	B13	*Cistenides hyperborea*	5	950	775
Future	Rothera	*Aequiyoldia eightsi*	1	44	70
Future	Rothera	*Aequiyoldia eightsi*	2	134	85
Future	Rothera	*Aequiyoldia eightsi*	3	145	47

Table S6(b)

Climate	Station	Species identity	Replicate	SBR (mm)	${}^{f\text{-}SPI}L_{median}$ (mm)	${}^{f\text{-}SPI}L_{max}$ (mm)	Δ[Br^-] (mg L^{-1})	Δ[NH_4-N] (lnRR)	Δ[NO_2-N] (lnRR)	Δ[NO_3-N] (lnRR)	Δ[PO_4-P] (lnRR)
Ambient	B13	*Astarte crenata*	1	18.864	2.955	19.659	-246.343	-0.699	-0.081	-0.102	0.136
Ambient	B13	*Astarte crenata*	2	23.222	4.556	22.111	-8.974	-0.976	0.006	-0.031	0.326
Ambient	B13	*Astarte crenata*	3	11.150	2.450	9.100	-76.436	-0.587	0.160	0.057	0.364
Ambient	B16	*Astarte crenata*	1	16.348	2.472	4.382	52.534	-0.140	-0.186	0.005	0.353
Ambient	B16	*Astarte crenata*	2	9.323	2.396	3.906	7.825	-0.963	0.503	0.295	0.475
Ambient	B16	*Astarte crenata*	3	28.309	3.382	10.515	61.468	-0.693	0.286	0.208	0.339
Ambient	B13	*Ctenodiscus crispatus*	1	11.979	4.375	20.990	-490.463	0.417	0.947	0.332	0.666
Ambient	B13	*Ctenodiscus crispatus*	2	8.688	4.750	21.688	-728.410	0.354	0.036	-0.130	0.048
Ambient	B13	*Ctenodiscus crispatus*	3	8.796	3.102	14.907	-74.363	0.046	0.066	-0.092	0.137
Ambient	B16	*Ctenodiscus crispatus*	1	8.827	1.888	12.245	-305.589	0.952	0.792	-0.320	0.059
Ambient	B16	*Ctenodiscus crispatus*	2	7.585	2.797	11.398	101.869	-0.466	-0.737	-0.762	-0.079
Ambient	B16	*Ctenodiscus crispatus*	3	9.158	5.211	24.053	-211.187	-0.505	-0.352	-0.170	0.492
Ambient	B13	*Cistenides hyperborea*	1	27.088	2.912	52.198	-268.773	-0.014	1.266	0.576	0.368
Ambient	B13	*Cistenides hyperborea*	2	27.767	2.136	67.670	146.754	-0.068	0.917	0.271	0.299
Ambient	B13	*Cistenides hyperborea*	3	26.429	3.929	82.976	336.853	-0.167	0.989	0.126	0.054
Ambient	Rothera	*Aequiyoldia eightsi*	1	11.183	1.613	11.129	596.093	-0.077	0.316	0.297	0.916
Ambient	Rothera	*Aequiyoldia eightsi*	2	10.562	1.966	5.899	-142.498	-0.311	0.194	0.183	0.926
Ambient	Rothera	*Aequiyoldia eightsi*	3	20.529	1.202	11.538	-1102.750	-0.048	0.222	0.474	2.781
Ambient	Rothera	*Laternula elliptica*	1	17.188	2.135	6.146	270.931	0.486	0.261	0.280	0.663
Ambient	Rothera	*Laternula elliptica*	2	18.177	2.396	6.927	-209.815	0.741	-0.623	-0.295	0.597
Ambient	Rothera	*Laternula elliptica*	3	12.832	2.345	4.381	100.282	-1.157	0.555	0.468	0.753
Future	B13	*Astarte crenata*	1	15.924	3.361	14.874	-486.898	-1.431	0.067	0.065	0.261
Future	B13	*Astarte crenata*	2	16.833	2.833	10.333	15.398	-0.217	0.050	-0.061	0.248
Future	B13	*Astarte crenata*	3	18.211	2.632	10.789	-114.633	0.199	-0.033	-0.101	0.204
Future	B16	*Astarte crenata*	1	18.211	2.737	5.211	699.360	-0.225	0.322	0.199	0.362

Future	B16	*Astarte crenata*	2	9.837	1.870	3.984	-52.708	-1.515	0.365	0.418	0.598
Future	B16	*Astarte crenata*	3	10.865	2.644	6.346	-270.559	-0.384	-0.123	-0.231	0.095
Future	B13	*Ctenodiscus crispatus*	1	10.891	3.168	15.941	163.403	0.540	0.482	0.025	0.337
Future	B13	*Ctenodiscus crispatus*	2	11.274	2.217	13.160	-282.644	0.355	-1.062	-0.533	0.150
Future	B13	*Ctenodiscus crispatus*	3	15.577	0.865	17.308	-309.883	-0.493	0.518	-0.042	0.348
Future	B16	*Ctenodiscus crispatus*	1	9.767	3.488	22.907	-769.134	0.073	0.673	-0.211	-0.076
Future	B16	*Ctenodiscus crispatus*	2	10.709	1.231	9.478	-894.046	0.817	1.386	0.121	0.475
Future	B16	*Ctenodiscus crispatus*	3	8.713	2.794	15.404	-1235.051	-0.099	1.258	0.062	0.333
Future	B13	*Cistenides hyperborea*	1	22.525	3.990	71.061	-417.178	0.004	0.944	-1.309	0.420
Future	B13	*Cistenides hyperborea*	2	36.031	4.072	84.691	-715.989	-0.101	0.703	-1.478	0.402
Future	B13	*Cistenides hyperborea*	3	20.850	3.400	68.700	-556.549	0.034	1.028	-0.962	0.907
Future	Rothera	*Aequiyoldia eightsi*	1	23.739	2.838	17.477	-955.795	-0.157	-0.737	-0.166	1.082
Future	Rothera	*Aequiyoldia eightsi*	2	13.611	1.852	18.194	260.590	-0.994	-0.179	-0.159	0.836
Future	Rothera	*Aequiyoldia eightsi*	3	22.120	2.826	17.174	115.246	-0.251	-0.653	-0.143	0.336
Future	Rothera	*Laternula elliptica*	1	7.718	1.845	6.262	-417.679	-1.732	-0.021	-0.079	0.303
Future	Rothera	*Laternula elliptica*	2	10.909	2.273	5.289	157.486	-0.367	-0.200	-0.282	0.168
Future	Rothera	*Laternula elliptica*	3	17.603	2.893	9.793	-102.498	0.593	0.358	0.197	0.465

References cited in electronic supplementary material

Barnes, DKA *et al.* 2019 Changing Arctic Ocean Seafloor JR18006 Cruise Report. Retrieved from https://www.bodc.ac.uk/resources/inventories/cruise_inventory/reports/jr18006.pdf

Blott, SJ, Pye K. 2001. GRADISTAT: a grain size distribution and statistics package for the analysis of unconsolidated sediments. *Earth Surface Processes and Landforms*, **26**(11), 1237–1248. (doi: 10.1002/esp.261)

Jørgensen L, Ljubin P, Skjoldal H, Ingvaldsen R, Anisimova N, Manushin I. 2014 Distribution of benthic megafauna in the Barents Sea: baseline for an ecosystem approach to management. *ICES Journal of Marine Science*, **72**, 595-613. (doi:10.1093/icesjms/fsu106)

Loeng H. 1991 Features of the physical oceanographic conditions of the Barents Sea. *Polar Research*, **10**(1), 5–18. (doi:10.1111/j.1751-8369.1991.tb00630.x)

Robbins LL, Hansen ME, Kleypas JA, Meylan SC. 2010 CO2calc: A User Friendly Carbon Calculator for Windows, Mac OS X and iOS (iPhone). *United States Geological Survey*, **1280**, 1210–1280. (doi:10.3133/OFR20101280)
